# Diversification of Secondary Metabolite Biosynthetic Gene Clusters Coincides with Lineage Divergence in *Streptomyces*

**DOI:** 10.3390/antibiotics7010012

**Published:** 2018-02-13

**Authors:** Mallory J. Choudoir, Charles Pepe-Ranney, Daniel H. Buckley

**Affiliations:** School of Integrative Plant Science, Bradfield Hall 705, Cornell University, Ithaca, NY 14853, USA; mjchoudoir@gmail.com (M.J.C.); chuck.peperanney@gmail.com (C.P.-R.)

**Keywords:** *Streptomyces*, biogeography, comparative genomics, diversification, secondary metabolite biosynthetic gene clusters, SMGC, natural products

## Abstract

We have identified *Streptomyces* sister-taxa which share a recent common ancestor and nearly identical small subunit (SSU) rRNA gene sequences, but inhabit distinct geographic ranges demarcated by latitude and have sufficient genomic divergence to represent distinct species. Here, we explore the evolutionary dynamics of secondary metabolite biosynthetic gene clusters (SMGCs) following lineage divergence of these sister-taxa. These sister-taxa strains contained 310 distinct SMGCs belonging to 22 different gene cluster classes. While there was broad conservation of these 22 gene cluster classes among the genomes analyzed, each individual genome harbored a different number of gene clusters within each class. A total of nine SMGCs were conserved across nearly all strains, but the majority (57%) of SMGCs were strain-specific. We show that while each individual genome has a unique combination of SMGCs, this diversity displays lineage-level modularity. Overall, the northern-derived (NDR) clade had more SMGCs than the southern-derived (SDR) clade (40.7 ± 3.9 and 33.8 ± 3.9, mean and S.D., respectively). This difference in SMGC content corresponded with differences in the number of predicted open reading frames (ORFs) per genome (7775 ± 196 and 7093 ± 205, mean and S.D., respectively) such that the ratio of SMGC:ORF did not differ between sister-taxa genomes. We show that changes in SMGC diversity between the sister-taxa were driven primarily by gene acquisition and deletion events, and these changes were associated with an overall change in genome size which accompanied lineage divergence.

## 1. Introduction

Microbial secondary metabolism encapsulates a remarkable diversity of natural products with an extensive range of biological activities. Secondary metabolites differ from primary metabolites in that they are not involved in essential catabolic and anabolic activities required for normal growth and reproduction, but may contribute significantly to an individual’s fitness [1]. While primary metabolic pathways are often conserved deeply within a phylogeny, secondary metabolic pathways are more divergent, often being species or strain-specific, with conservation sometimes observed among closely related species and genera [2]. This phylogenetic pattern suggests an adaptive role for secondary metabolites, and if secondary metabolism pathways provide adaptive benefits, their evolution might drive or reinforce evolutionary processes that result in microbial diversification and speciation [3].

The values of natural products to humanity are widely recognized, yet because most research has focused on their discovery and human-centric relevance, we are still far from understanding their biological role in natural systems. The discovery and application of antibiotics revolutionized medicine in the 1940’s, sparking the “golden age” of antibiotics between 1950 and 1960, during which time approximately half of the microbial-derived drugs we use today were discovered [4]. Presently, thousands of bioactive compounds with antibacterial, antifungal, and antitumor activities are cataloged [5,6], and yet these represent only a fraction of actual natural product diversity [7]. In addition, microbial populations in situ are exposed to natural products at concentrations far below the lethal clinical dose, and hence these compounds may serve different functions in the environment from those observed during therapeutic application. We know that secondary metabolites can mediate diverse biotic interactions including mutualistic interactions, competition for nutrients, metal scavenging, and plant-microbe and insect-microbe symbioses [8,9,10], which can all have profound impacts on microbial fitness. It is clear that natural products must have considerable impacts on microbial ecology and evolution and that understanding the biology and evolutionary history of natural products will enhance our ability to use these agents therapeutically.

Soil-dwelling actinomycetes are the predominant source of microbial-derived therapeutic natural products, and the majority of described bioactive compounds originate from the genus *Streptomyces* [6,7]. The *Streptomyces* life cycle resembles that of many fungi, consisting of filamentous growth, formation of mycelia, and production of aerial hyphae and spores. Indeed, *Streptomyces* were thought to be an intermediary between bacteria and fungi until as recently as the 1950’s [11]. However, *Streptomyces* are Gram-positive *Actinobacteria* with long linear chromosomes that have a high G+C content [12]. Traditionally, *Streptomyces* species are often known to produce several secondary metabolites when grown in culture. Genome sequencing, however, reveals that *Streptomyces* contain an enormous reservoir of “cryptic secondary metabolites” which are not expressed under standard laboratory conditions [13]. For instance, while *Streptomyces coelicolor* A3(2) was known to produce several well characterized secondary metabolites, genome sequencing discovered that it actually contained >20 biosynthetic gene clusters not expressed when grown in culture [14]. Genes within secondary metabolite biosynthetic gene clusters (SMGCs) are co-localized as operons within discrete genomic regions. SMGCs have recognizable functional domains, so SMGCs are readily predicted using bioinformatics [15]. Phylogenetic conservation of SMGCs between closely related microbes suggests that these secondary metabolites may have ecological roles which facilitate microbial diversification [16,17,18].

The evolutionary and ecological processes that govern SMGC diversity remain largely unexplored. The richness of SMGCs within soils is linked to both edaphic and biotic factors [19,20]. For example, the production of antibiotics by *Streptomyces* isolated from prairie soils is highly variable between strains and correlates poorly with 16S rRNA gene phylogeny, suggesting a role of selection acting at small spatial scales [21,22]. Conversely, at larger spatial scales, *Streptomyces* SMGC composition varies in relation to both spatial distance and environmental dissimilarity [23]. Furthermore, evidence within *Streptomyces* for endemism at inter-continental and regional geographic scales [16,24,25] suggests limits to dispersal at large spatial scales. These data indicate that both adaptive and neutral processes contribute to patterns of SMGC biogeography.

Microbial biogeography is readily explored with geographically explicit microbial culture collections (reviewed in [26]), and the genus *Streptomyces* is an ideal model system to evaluate the influence of SMGC dynamics on patterns of diversification. We previously assembled a culture collection of *Streptomyces* from sites spanning the United States, and we observed evidence for dispersal limitation, as well as a latitudinal gradient of species riches and intraspecific nucleotide diversity [27,28]. From this culture collection, we have identified *Streptomyces* sister-taxa that have geographic ranges delimited by latitude and have patterns of gene flow and genomic diversity consistent with their diversification from a recent common ancestor [28,29]. Here, we evaluate changes in SMGC diversity between these *Streptomyces* sister-taxa to explore SMGC evolutionary dynamics during the divergence of *Streptomyces* species.

## 2. Results and Discussion

### 2.1. Genomic Divergence between Streptomyces Sister-Taxa

**Figure 1:**
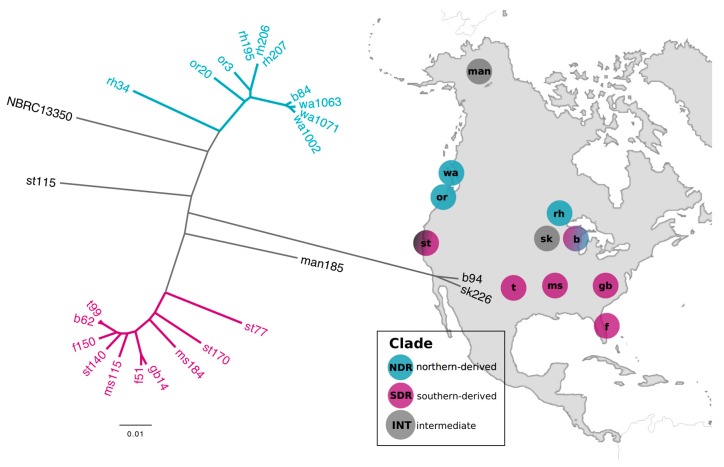
The northern-derived (NDR) and southern-derived (SDR) clades are closely related sister-taxa and yet were isolated from soils of different latitude. The un-rooted tree was constructed from multiple whole genome alignments with maximum likelihood and a GTRGAMMA model of evolution. Scale bar represents nucleotide substitutions per site. Colored branches depict the northern-derived (NDR) and southern-derived (SDR) clades. Strain names reflect the sample site they were isolated from (Table S1). Genome NBRC 13350 is the publically available type strain *Streptomyces griseus* subsp. *griseus* NBRC 13350. Sample locations are shown in the right panel and labeled with the site code. Circles are colored to reflect the geographic distribution of clades. (Figure modified from [29]).

We used comparative genomics to analyze patterns of genomic diversity and SMGC content in 24 *Streptomyces* representing sister-taxa and related strains. These strains were identified through a phylogenetic analysis of a *Streptomyces* culture collection [28] generated from soils of ecologically similar grassland sites spanning 6000 km across the continental United States (Figure 1, Table S1). The sister-taxa, which we have designated the northern-derived (NDR) and southern-derived (SDR) clades, were defined by their geographic range and genomic similarity (Figure 1). Each clade contains ten isolates, and an additional four genomes represent intermediate (INT) taxa.

Assembled genomes are 7.5–9.1 Mb with a G+C content of 71.4–72.5% and 6776–8078 predicted open reading frames (ORFs) (Table S2). The core gene content across all 24 strains is comprised of 3234 orthologous genes (representing 2778 single-copy genes), with a total of 22,054 genes in the overall pan-genome. All isolates affiliate taxonomically with the *Streptomyces griseus* species cluster [30] and share >90% average nucleotide identity (ANI) with the type strain *Streptomyces griseus* subsp. *griseus* NBRC 13350 (Figure 1).

The NDR core genome is comprised of 4234 genes, and the SDR core genome is comprised of 4400 genes. The NDR and SDR clades share a recent phylogenetic ancestor and have nearly identical 16S rRNA genes (inter-lineage nucleotide dissimilarity of 0–0.21% between strains). Strains within each clade have a whole genome ANI value ranging from 95.6% to 99.9%, while the ANI between strains of NDR and SDR range from 92.6% to 93.3% (Figure 1). Distinct microbial species are typically distinguished by ANI in the range of 95–96% [31]. Comparative population genomics reveals signatures of genomic differentiation and gene flow limitation between NDR and SDR consistent with expectations of allopatric diversification [29]. Collectively, these results indicate that NDR and SDR clades represent distinct microbial species which have recently diverged from a common ancestor.

### 2.2. Secondary Metabolite Biosynthetic Gene Cluster (SMGC) Identification and Classification

**Figure 2:**
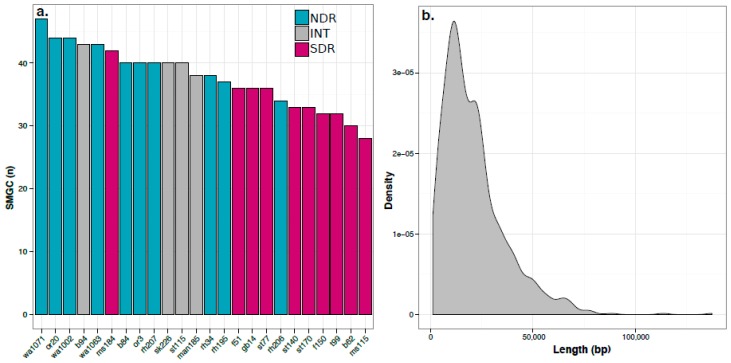
NDR strains have more secondary metabolite biosynthetic gene clusters (SMGCs) than SDR strains (*t*-test, *p* < 0.001). (**a**). Bars indicate the number of SMGCs identified in each genome and are colored according to clade affiliation, and genome names reflect the site of isolation as identified in Table S1; (**b**). Kernal density plot shows the distribution of SMGC length (bp).

We used antiSMASH [32] to identify SMGCs in the genomes of our *Streptomyces* sister-taxa. To assess the novelty of these SMGCs, we utilized antiSMASH’s downstream annotation pipeline, which annotates SMGCs based on similarity to genes and pathways present within the Minimum Information about a Biosynthetic Gene cluster (MIBiG) database. The antiSMASH pipeline annotated 120 SMGCs across the 24 strains (Table S3). Each genome had between 28 and 47 SMGCs which ranged in size from 1 to 137 Kb (20.9 ± 15.7 Kb, mean ± S.D., respectively) (Figure 2). This range in SMGC content is consistent with the results obtained from previous genomic surveys of *Streptomyces* [14,33,34,35,36]. The NDR clade has a greater number of SMGCs per genome than the SDR clade (40.7 ± 3.9, 33.8 ± 3.9, mean ± S.D., respectively; *t*-test, *p* < 0.001; Figure 2a). The NDR clade also has a greater number of ORFs per genome than the SDR clade (7775 ± 196 and 7093 ± 205, mean and S.D., respectively; *t*-test, *p* < 0.001; Table S2). Correspondingly, NDR strains also have larger genomes than SDR strains (8.7 ± 0.25 Mb and 7.9 ± 0.21 Mb, mean ± S.D., respectively; *t*-test, *p* < 0.001; Table S2). We observed a strong positive correlation between genome size and number of SMGCs across all genomes examined (Pearson’s *r* = 0.66, *p* < 0.001). 

Only 21% (*n* = 25) of the MIBiG-annotated SMGCs represent well-characterized biosynthetic gene clusters (in which ≥70% of the genes in a SMGC show similarity to genes within the most similar known cluster from the MIBiG database) (Table S3). In addition, each genome harbors five to 25 potentially novel SMGCs with low similarity to biosynthetic pathways within the MIBiG database. These findings indicate that the diversity of *Streptomyces* SMGCs found within public databases remains low and that a vast reservoir of *Streptomyces* SMGC diversity remains to be characterized within natural populations.

**Figure 3:**
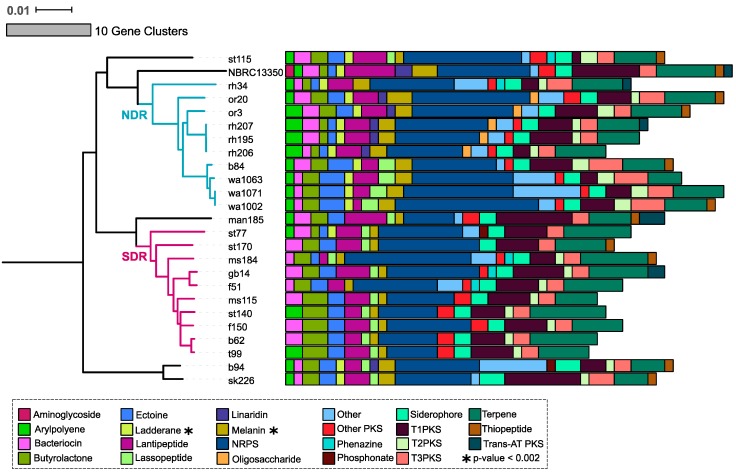
A total of 22 SMGC classes were observed in NDR and SDR genomes by antiSMASH [32]. The tree reflects phylogenetic relationships between *Streptomyces* sister-taxa genomes and was constructed from multiple whole genome alignments (see Figure 1). Scale bar represents nucleotide substitutions per site. Tree branches are colored according to clade affiliation. Bars depict the number of gene clusters belonging to each class for each genome. Colors illustrate gene cluster class as provided by the legend. Asterisks note gene cluster classes that are significantly enriched between clades (*t*-test and Bonferonni correction for multiple comparisons, *p* < 0.002).

The SMGCs predicted by antiSMASH within our *Streptomyces* sister-taxa encompass 22 classes of natural products. Most of these classes, including bacteriocin, butyrolactones, ectoine, lantipeptide, melanin, non-ribosomal peptide synthases (NRPS), siderophore, polyketide synthases (PKS), and terpene gene clusters, are widely conserved at the genus level [2]. The most abundant SMGC classes in our genomes are NRPS and terpene clusters (Figure 3, Table S3). Many of the predicted gene clusters are NRPS-PKS hybrids (Table S3). Given the similar structure and activity between NRPS and PKS [37], it is unsurprising that hybrid NRPS-PKS clusters are commonly detected in *Streptomyces* genomes [38,39]. Most SMGC classes are present in both NDR and SDR clades, but the relative abundance of each class differs between genomes, as well as between clades (Figure 3). We observe the significant enrichment of melanin and ladderane gene clusters in NDR compared to SDR (*t*-test with Bonferrori correction, *p* < 0.002). Additionally, NDR genomes harbor linaridin gene clusters, which are entirely absent from SDR genomes (Figure 3) but are found in the type strain *Streptomyces griseus* NBRC subsp. *griseus* 13350 [40]. Interestingly, antiSMASH did not identify aminoglycoside biosynthetic clusters in our *Streptomyces* isolates, and all of these genomes presumably lack genes for streptomycin biosynthesis (Figure 3). Schatz and Waksman reported the isolation of streptomycin from *Streptomyces griseus* in 1944, and this was the first antibiotic used to successfully combat tuberculosis [41]. However, not all *Streptomyces griseus* isolates produce streptomycin [42,43].

### 2.3. Core and Accessory SMGCs of Streptomyces Sister-Taxa

**Figure 4:**
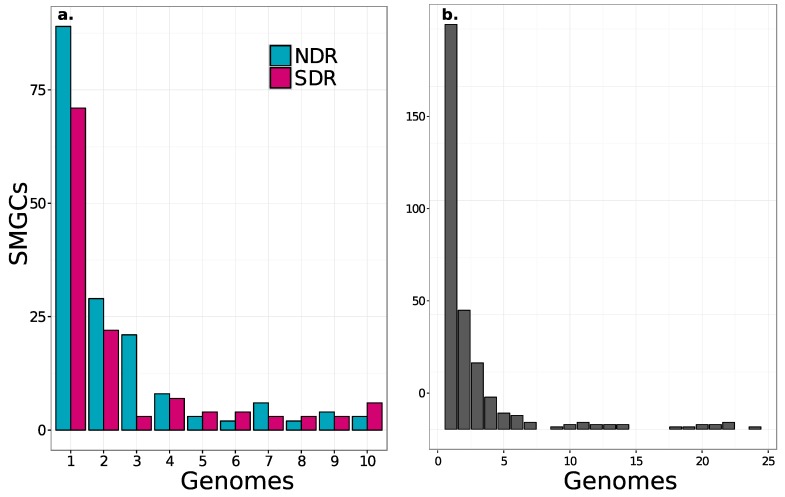
The frequency distribution of SMGCs across strains shows that most SMGCs are strain-specific and fewer are species-specific. Results are shown both for NDR and SDR. (**a**) and for all 24 genomes; (**b**). Non-redundant orthologous SMGCs were defined using our annotation-independent approach (see Materials and Methods).

**Figure 5:**
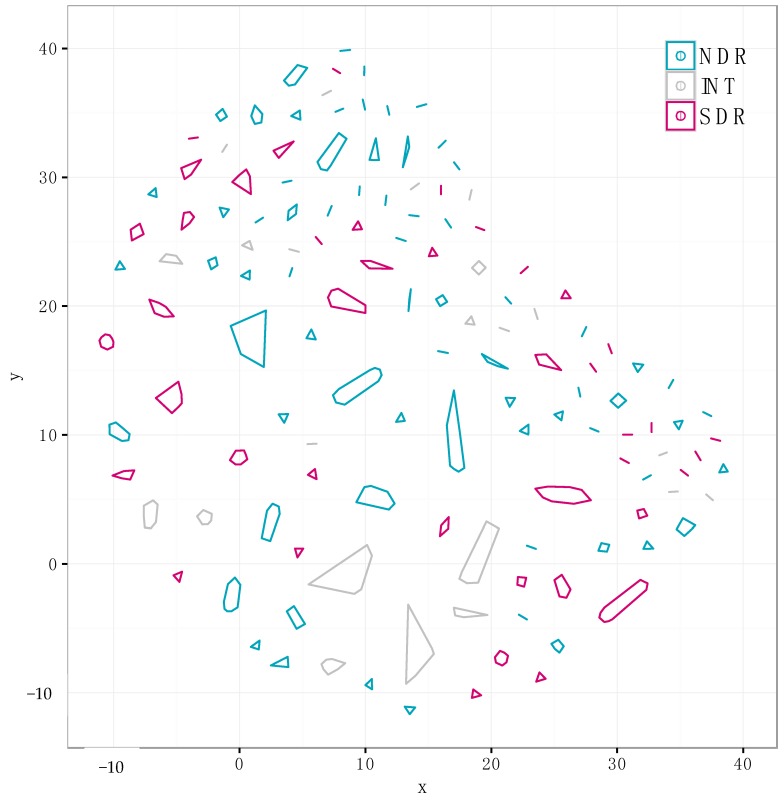
We identified 310 non-redundant distinct SMGCs using our annotation-independent gene clustering approach (see Materials and Methods). Each point represents a unique SMGC from a single genome, and colors correspond to clade affiliation. SMGCs with a similar gene composition are clustered spatially, and cluster membership is depicted with polygons. The same data is presented in a different network diagram in Figure S1.

**Figure 6:**
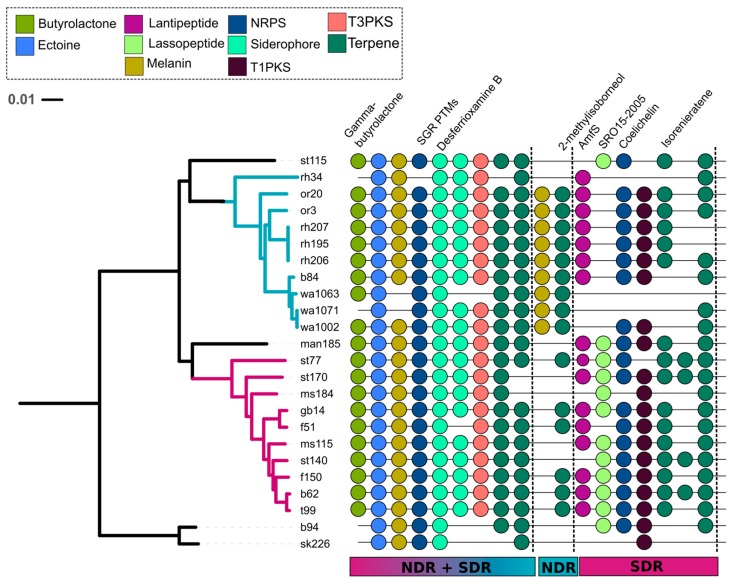
A total of nine core (i.e., conserved in ≥80% of genomes) SMGCs were found in both NDR and SDR. The NDR clade had 11 core SMGCs and the SDR clade had 15 core SMGCs. The tree reflects phylogenetic relationships between *Streptomyces* sister-taxa genomes and was constructed from multiple whole genome alignments (see Figure 1). Scale bar represents nucleotide substitutions per site. Tree branches are colored according to clade affiliation. Core orthologous SMGCs (depicted by colored circles) were determined using the antiSMASH [32] MIBiG annotation pipeline or were defined using our annotation-independent approach (see Materials and Method). Colors correspond to SMGC class (see legend), and natural product annotations are labeled if available.

Comparative population genomics and pan-genome analyses can offer powerful insights into the processes underlying species divergence [44,45]. Given that many of our SMGCs have low similarity to biosynthetic pathways in public databases, we determined shared orthologous SMGCs within our genomes using an annotation-independent approach that compares SMGCs based on similarity in nucleotide composition and gene content (see Materials and Methods). This approach identified 310 non-redundant SMGCs within the pan-genome of all 24 strains (Figure 4 and Figure 5); this number is greater than the number of MIBiG-annotated SMGCs because it classified both known and unknown pathways into distinct non-redundant gene clusters. Only two SMGCs are conserved in all 24 genomes, an ectoine gene cluster and the siderophore desferrioxamine B (Figure 6). Desferrioxamine siderophores are commonly observed in other species of *Streptomyces* and acintomycetes [46,47].

We observed that core SMGC content increased with phylogenetic similarity, but that more than half of the SMGCs were strain-specific (Figure 4 and Figure 5). NDR and SDR shared nine core SMGCs (present in ≥80% of genomes), while NDR strains shared 11 core SMGCs (nine in the conserved core and two in the NDR-specific core), and SDR strains shared 15 core SMGCs (nine in the conserved core and six in the SDR-specific core) (Figure 6). In addition, there were 158 accessory SMGCs (present in <80% genomes) in NDR and 114 accessory SMGCs in SDR (Figure 4). Most SMGCs were observed at low to intermediate frequencies (Figure 4 and Figure 5), and 177 SMGCs were strain-specific, with each *Streptomyces* genome harboring one to 19 exclusive SMGCs. These estimates are generally consistent with previous observations that indicate each different *Streptomyces* species will harbor a distinct repertoire of natural product pathways [17]. For example, Seipke [36] estimated 18 core SMGCs for six *Streptomyces albus* isolates. However, despite the phylogenetic conservation of core SMGC content, even *Streptomyces* with identical 16S rRNA gene sequences can have distinct secondary metabolite profiles [48], indicating that SMGC content exhibits significant strain to strain variability within a species. Thus, we propose that core SMGCs reflect the shared evolutionary history of *Streptomyces* genomes, while patterns of the accessory SMGC carriage suggest lineage and strain-specific processes across more recent evolutionary time scales. 

### 2.4. Evolutionary Dynamics of Core and Accessory SMGCs

To address potential lineage-specific mechanisms of divergence, we next evaluated the evolutionary dynamics of SMGCs. Most shared SMGCs occur within rather than between clades (Figure 5 and Figure S1). A total of 78 SMGCs are shared among two or more NDR genomes, and 55 are shared among SDR genomes, but only 37 SMGCs are shared across clade boundaries (i.e., found in both NDR and SDR genomes). Furthermore, network analysis reveals unique patterns of SMGC sharing that manifests as nodes of connectivity within clades (Figure S1). This network indicates that there is a core set of SMGC content which links NDR and SRD and which must be ancestral, that there is a clade-specific core set of SMGCs which link the strains of each clade together based on shared SMGC content, and that there are a large number of strain-specific SMGCs (Figure 5 and Figure S1).

Differences in gene content between closely related microbes ultimately result from gene gain and loss events [49,50,51]. Although deletion bias is strong in bacterial genomes [52], gene acquisitions can drive rapid genome innovation and evolution [53]. Gene clusters are often acquired through horizontal gene transfer leading to the formation of new operons in bacterial genomes [54], and many SMGCs in actinomycetes are believed to be the result of horizontal gene transfer [16,18,33,55]. Parsimony predicts that low frequency and strain-specific genes are likely the result of a recent acquisition, while high frequency “near core” genes are the likely result of recent deletion events [56]. Hence, we are able to infer SMGC gain and loss dynamics in our *Streptomyces* sister-taxa from SMGC frequency distributions (Figure 4). 

**Figure 7:**
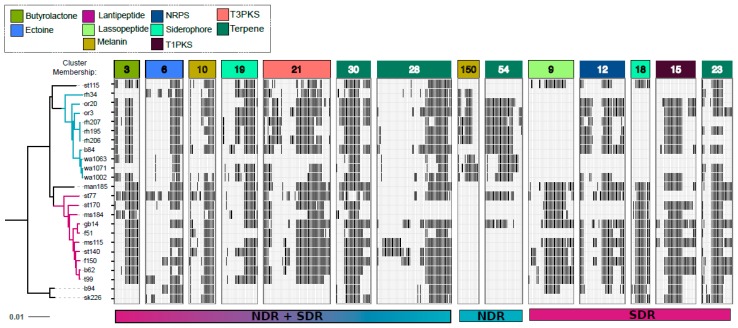
Gene content of core SMGCs vary within and between clades as a result of gene acquisition and deletion events. Panels depict the gene content (i.e., genetic architecture) of core SMGCs (i.e., conserved in ≥80% of genomes), the NDR-specific SMGC core, and the SDR-specific SMGC core. Black bars within the panels represent orthologous genes. The tree reflects phylogenetic relationships between *Streptomyces* sister-taxa genomes and was constructed from multiple whole genome alignments (see Figure 1). Scale bar represents nucleotide substitutions per site. Panel colors correspond to SMGC class (see legend). Panels are labeled with the SMGC cluster membership (see Table S3) defined using our annotation-independent approach (see Materials and Methods).

The majority of SMGCs observed within the sister-clades occurred in only one or a few strains, and this suggests that gene acquisition is a major force that drives the diversity of SMGC pathways in *Streptomyces*. However, each clade has a distinct set of core and accessory SMGCs (Figure 3, Figure 5 and Figure 6 and Figure S1), and this suggests that SMGC composition (Figure 7) may underlie ecological traits that promote or reinforce lineage divergence. For example, nearly all genomes within the NDR clade (with the exception of rh34) harbor a melanin gene cluster which is absent from both the intermediate (INT) and SDR genomes, suggesting that horizontal gene transfer of the melanin gene cluster into the immediate ancestor of NDR accompanied lineage divergence (Figure 6 and Figure 7). Overall, NDR has more low frequency SMGCs (present in one to three strains) than SDR (139 and 96, respectively) (Figure 4). This result suggests a greater rate of gene acquisition in NDR than in SDR and is consistent with the observation that NDR has more SMGCs (Figure 2) and larger genomes overall than SDR. While this difference in gene content is potentially adaptive, it could also be explained as a consequence of neutral demographic processes such as genome surfing (reviewed in [57]). However, the distribution of SMGC frequencies does not differ significantly between clades (Kolmogorov-Smirnov test, *p* = 0.4). Hence, while it seems clear that gene acquisition is a major driver of SMGC biodiversity, the role of gene acquisition in driving lineage divergence remains unclear.

We also see evidence that NDR has undergone the deletion of SMGC-associated genes inherited from the common ancestor of NRD and SDR. For example, the SRO15-2005 lassopeptide gene cluster is conserved in SDR and found in INT but absent from NDR, suggesting that deletion of this lassopeptide accompanied NDR divergence (Figure 6 and Figure 7). We also find that core SMGC gene loss is more common in NDR than SDR (strain-level deletions occur in six out of nine core gene clusters within NDR and two out of nine core gene clusters in SDR) (Figure 6). Similarly, we can observe SDR species-specific core gene clusters (AmfS, coelichelin, a T1PKS, and a terpene) that are found in only 70% (i.e., near core) of NDR strains (Figure 6). This pattern suggests that these SMGCs were present in the common ancestor of the two clades and subsequently deleted from NDR isolates. In addition, the butyrolactone operon (cluster 3) is comprised of more genes in SDR than in NDR, and this likely indicates active gene loss within this pathway for NDR strains (Figure 7). 

Taken together, these results suggest that the sister-clades are under different evolutionary pressures which drive dissimilarity in SMGC composition. NDR genomes have increased in size relative to their ancestors suggesting an overall increase in the rate of gene acquisition via horizontal gene exchange, and this increase in gene acquisition has resulted in an increase in strain-specific SMGC content in NDR. In addition, the presence of NDR-specific core SMGCs (e.g., melanin gene cluster) indicates that some horizontally acquired SMGC have gone to fixation within NDR. At the same time, deletion events in NDR have pruned away SMGCs inherited from ancestral lineages (i.e., those clusters present in SDR and INT). We hypothesize that these changes in SMGC content are likely to have effects on fitness which should act to reinforce lineage divergence either as a result of antagonism or niche differentiation.

## 3. Materials and Methods

### 3.1. Streptomyces Isolation and DNA Extraction

We built a culture collection of >1000 *Streptomyces* isolated from grassland soils (pH 3.9–7.3) sampled at 0–5 cm from sites across the United States [27]. Pure *Streptomyces* cultures were obtained from air-dried soils on glycerol-arginine agar (pH 8.7) containing antifungals as previously described [58]. Genomic DNA was extracted using a standard phenol/chloroform/isoamyl alcohol protocol from liquid cultures grown in yeast extract-malt extract medium (YEME) with 0.5% glycine [5] for 72 h shaking at 30 °C.

### 3.2. Whole Genome Sequencing, Assembly, and Annotation

*Streptomyces* genomic sequencing libraries were prepped with the Nextera DNA Library Preparation Kit (Illumina, San Diego, CA, USA), and draft genomes were generated using the Illumina HiSeq2500 platform (Illumina, San Diego, CA, USA) and paired-end 2 × 100 bp reads at the Cornell University Biotechnology Resource Center (BRC). Quality control and assembly was performed with the A5 pipeline [59], and genomes were annotated using the online RAST Server [60]. Multiple whole genome alignments were obtained with Mugsy [61], and trimAL v1.2 removed poorly aligned regions [62]. Orthologous genes were identified using ITEP [63] with MCL clustering parameters as follows: inflation value = 2.0, cutoff = 0.04, maxbit score. Average nucleotide identity (ANI) was determined using mother [64]. Genome sequences are available at NCBI under BioProject ID PRJNA401484 accession numbers SAMN07606143–SAMN07606166.

### 3.3. Phylogenetic Reconstruction

The phylogenetic relationship between genomes was reconstructed from DNA sequences of multiple whole genome alignments using maximum likelihood (ML) with the generalized time reversible nucleotide substitution model [65] with gamma distributed rate heterogeneity among sites (GTRGAMMA) in RAxML v7.3.0 [66]. Bootstrap support was determined using the RAxML rapid bootstrapping algorithm [67].

### 3.4. Secondary Metabolite Biosynthetic Gene Cluster (SMGC) Identification

Secondary metabolite biosynthetic gene clusters (SMGC) were predicted and annotated using the online server antiSMASH 3.0 [32]. We also used an annotation-independent approach to identify SMGCs shared between genomes. For each SMGC identified by antiSMASH, we used Prodigal [68] to call open reading frames (ORFs) and Parasail with default parameters to identify orthologous genes and orthologous gene groups [69]. We used the R package igraph [70] to cluster similar SMGCs, define cluster membership, and thus determine which SMGCs are shared between genomes. Cluster membership was determined based on gene content using a binary (i.e., Jaccard) dissimilarity distance of ≤4.0 generated from an orthologous group presence/absence table. Dissimilarity distances of >4.0 did not result in an appreciable gain in the number of total clusters. The SMGC network was visualized and analyzed with Cytoscape 3.3.0 [71].

## 4. Conclusions

We used comparative genomics to examine SMGC diversity within strains of two closely related *Streptomyces* species that recently diverged from a common ancestor. Our objective was to observe and explore the evolutionary dynamics of SMGCs that accompany evolutionary diversification and to assess SMGC conservation within and between closely related species. It is clear that gene gain and loss events drive major differences in SMGC composition, both within and between species. While both species share conserved core SMGCs, each clade has its own species-specific SMGC core, and the majority of SMGCs were strain-specific. This pattern indicates that these SMGCs, not present in shared ancestors, were acquired recently due to horizontal gene exchange. 

In addition, we observe that SMGCs that have been inherited from a shared ancestor can vary considerably in gene content, both due to the acquisition and deletion of individual genes within each gene cluster. We observe SMGC gain and loss dynamics that differ between clades and identify SMGC acquisition and deletion events that correspond to ancestral diversification events. These findings show that SMGC modification is associated with lineage divergence, though whether these changes cause or reinforce divergence directly or are an indirect product of evolutionary divergence remains to be seen. A limitation of the comparative genomics approach is that we cannot assess the ecological activity of a pathway from genome sequence data. It is possible that some (or all) of the strain-specific pathways, if acquired by recent horizontal exchange, may be non-functional. It is also possible that changes in SMGC architecture and gene content could alter pathway functionality and that pathways deemed orthologous on the basis of genetic similarity may have different functions in different strains.

Finally, we can conclude that, while strains within a species will share a core set of SMGCs, the number of accessory SMGC within a given species can be quite large, with each strain having its own repertoire of strain-specific SMGCs. Furthermore, the majority of these strain-specific SMGCs remain uncharacterized and lack similarity to SMGCs documented in public databases.

## Supplementary Materials

The following are available online at http://www.mdpi.com/2079-6382/7/1/12/s1, Figure S1: Each clade has a distinct SMGC network. The network illustrates inter- and intra-clade sharing of SMGC content. Large circles represent the genomes of Streptomyces strains and are labeled with isolate names and colored according to clade affiliation. Smaller circles represent non-redundant distinct SMGCs identified using our annotation-independent approach (see Materials and Methods). Lines connect each SMGC to the strains in which they are found. Network nodes and edges are scaled in proportion to the number of connections and colored according to gene cluster class (see legend). Network is arranged in the organic layout using Cytoscape 3.3.0 [71]. Core SMGCs can be observed as larger central nodes while strain specific and low frequency SMGCs occur around the edges of the graph; Table S1: The 24 Streptomyces genomes were isolated from 11 sites. Isolate names begin with the site code from which they were isolated from followed by strain number; Table S2: Genome and assembly characteristics for 24 Streptomyces genomes. The clade affiliations include the northern-derived (NDR), southern-derive (SDR), and intermediate (INT). Sample site of each isolate can be found in Table S1. Values report assembled draft genome size, genome-wide G+C content, the number of predicted open reading frames (ORFs), and the number of predicted secondary metabolite biosynthetic gene clusters (SMGCs) per genome; Table S3: SMGCs are predicted by antiSMASH [32] in our 24 Streptomyces genomes. For each SMGC, columns report the affiliated genome, clade, gene cluster class (hybrids are indicated by hyphens), gene cluster length (bp), natural product annotation provided by antiSMASH, cluster membership (Clust Memb), MIBiG database identification, the portion of genes with similarity to genes within the most similar known cluster from the MIBiG database (% Genes w/Similarity). Cluster membership was determined using our annotation-independent approach (see Materials and Methods). NA indicates information is not available.
