# Supplementary material for: Diversification of Secondary Metabolite Biosynthetic Gene Clusters Coincides with Lineage Divergence in Streptomyces
Source: Antibiotics (Basel). 2018 Feb 13;7(1):12. doi: 10.3390/antibiotics7010012 (PMC5872123; doi:10.3390/antibiotics7010012)
Supplement: Supplementary file 1 [file antibiotics-07-00012-s001.docx]

**Supplementary Materials**


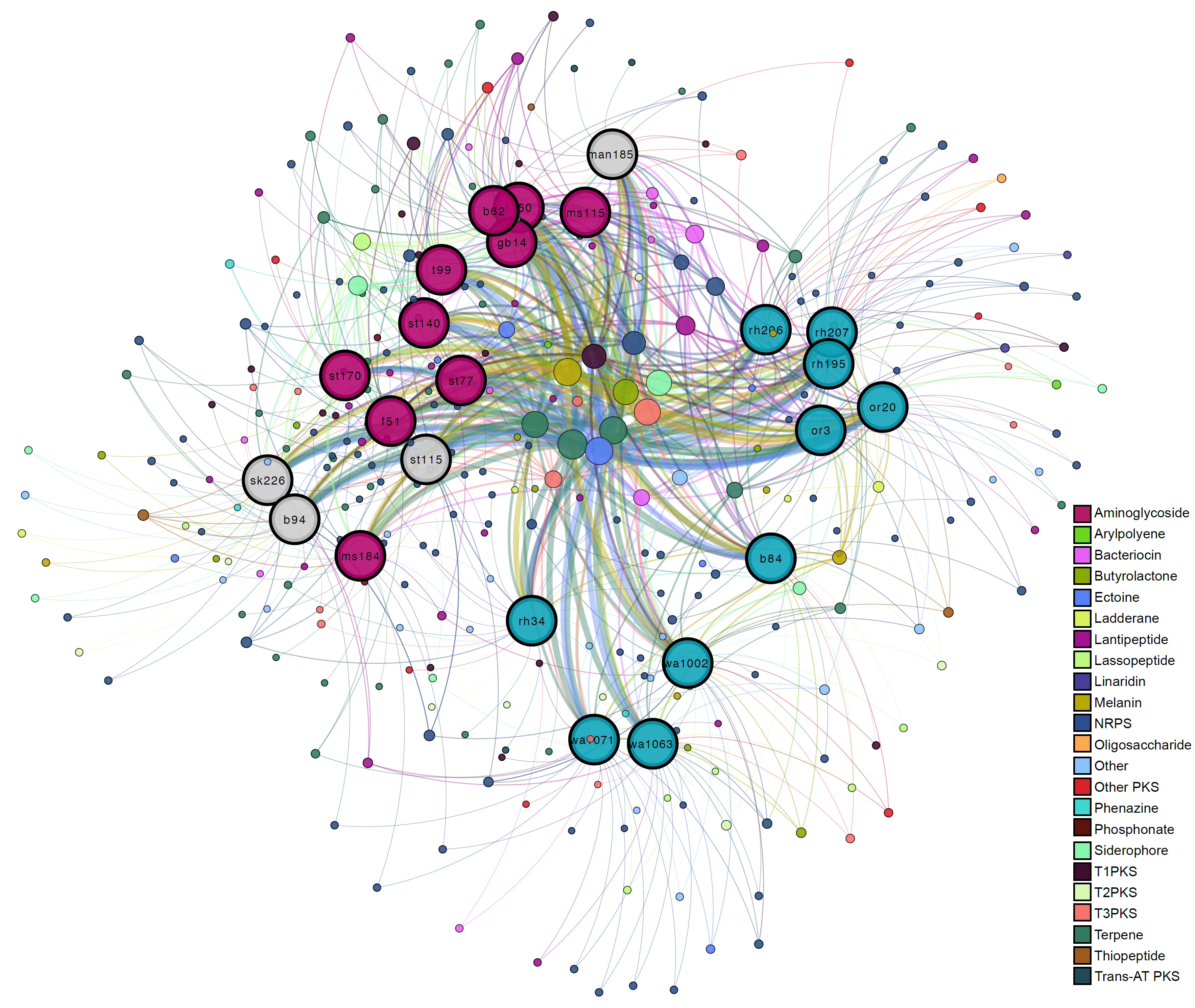


**Figure S1.** Each clade has a distinct SMGC network. The network illustrates inter- and intra-clade sharing of SMGC content. Large circles represent the genomes of *Streptomyces* strains and are labeled with isolate names and colored according to clade affiliation. Smaller circles represent non-redundant distinct SMGCs identified using our annotation-independent approach (see Materials and Methods). Lines connect each SMGC to the strains in which they are found. Network nodes and edges are scaled in proportion to the number of connections and colored according to gene cluster class (see legend). Network is arranged in the organic layout using Cytoscape 3.3.0 [[71]](https://paperpile.com/c/RiEeSd/Cuxt). Core SMGCs can be observed as larger central nodes while strain specific and low frequency SMGCs occur around the edges of the graph.

**Table S1.** The 24 *Streptomyces* genomes were isolated from 11 sites. Isolate names begin with the site code from which they were isolated from followed by strain number.

| **Site** | **Code** | **Latitude** | **Longitude** | **pH** |
| --- | --- | --- | --- | --- |
| Manley Hot Springs, AK | man | 63.87˚N | -149.02˚W | 5 |
| Bothell, WA | wa | 47.73˚N | -122.24˚W | 5.3 |
| Astoria, OR | or | 46.18˚N | -123.85˚W | 3.9 |
| Rhinelander, WI | rh | 45.57˚N | -89.33˚W | 6.5 |
| Bear Creek, WI | sk | 43.35˚N | -90.1˚W | 5.7 |
| Brookfield, WI | b | 43.06˚N | -88.13˚W | 6.8 |
| Palo Alto, CA | st | 37.43˚N | -122.17˚W | 7.1 |
| Greensboro, NC | gb | 36.09˚N | -79.89˚W | 5.6-7.0 |
| Starkville, MS | ms | 33.46˚N | -88.8˚W | 6.8 |
| Austin, TX | t | 30.2˚N | -97.77˚W | 7.3 |
| Fort Pierce, FL | f | 27.54˚N | -80.35˚W | 7.1 |

**Table S2.** Genome and assembly characteristics for 24 *Streptomyces* genomes. The clade affiliations include the northern-derived (NDR), southern-derive (SDR), and intermediate (INT). Sample site of each isolate can be found in Table S1. Values report assembled draft genome size, genome-wide G+C content, the number of predicted open reading frames (ORFs), and the number of predicted secondary metabolite biosynthetic gene clusters (SMGCs) per genome.

| **Genome** | **Clade** | **Size (Mb)** | **G+C %** | **ORFs** | **SMGCs** |
| --- | --- | --- | --- | --- | --- |
| b62 | SDR | 7.8 | 71.6 | 7073 | 30 |
| b84 | NDR | 8.7 | 71.5 | 7657 | 40 |
| b94 | INT | 8 | 72.5 | 6939 | 43 |
| f150 | SDR | 8.1 | 71.6 | 7320 | 32 |
| f51 | SDR | 7.7 | 71.7 | 6851 | 36 |
| gb14 | SDR | 7.9 | 71.6 | 6998 | 36 |
| man185 | INT | 8.1 | 71.6 | 7244 | 38 |
| ms115 | SDR | 7.5 | 71.7 | 6776 | 28 |
| ms184 | SDR | 7.9 | 71.7 | 6958 | 42 |
| or20 | NDR | 9.1 | 71.5 | 8087 | 44 |
| or3 | NDR | 8.9 | 71.7 | 7956 | 40 |
| rh195 | NDR | 8.6 | 71.5 | 7804 | 37 |
| rh206 | NDR | 8.6 | 71.5 | 7817 | 34 |
| rh207 | NDR | 8.6 | 71.5 | 7825 | 40 |
| rh34 | NDR | 8.2 | 71.5 | 7392 | 38 |
| sk226 | INT | 8 | 72.5 | 6930 | 40 |
| st115 | INT | 8.4 | 71.7 | 7498 | 40 |
| st140 | SDR | 8 | 71.5 | 7184 | 33 |
| st170 | SDR | 8.1 | 71.4 | 7351 | 33 |
| st77 | SDR | 8.2 | 71.5 | 7346 | 36 |
| t99 | SDR | 7.8 | 71.6 | 7076 | 32 |
| wa1002 | NDR | 8.6 | 71.5 | 7577 | 44 |
| wa1063 | NDR | 8.9 | 71.4 | 7879 | 43 |
| wa1071 | NDR | 8.8 | 71.4 | 7755 | 47 |

**Table S3.** SMGCs are predicted by antiSMASH [[32]](https://paperpile.com/c/RiEeSd/507A) in our 24 *Streptomyces* genomes. For each SMGC, columns report the affiliated genome, clade, gene cluster class (hybrids are indicated by hyphens), gene cluster length (bp), natural product annotation provided by antiSMASH, cluster membership (Clust Memb), MIBiG database identification, the portion of genes with similarity to genes within the most similar known cluster from the MIBiG database (% Genes w/ Similarity). Cluster membership was determined using our annotation-independent approach (see Materials and Methods). NA indicates information is not available.

| **Genome** | **Clade** | **Class** | **Length (bp)** | **Natural Product** | **Clust Memb** | | **MIBiG ID** | **% Genes w/ Similarity** |
| --- | --- | --- | --- | --- | --- | --- | --- | --- |
| b62 | SDR | Bacteriocin | 11373 | NA | 1 | NA | | NA |
| b62 | SDR | Bacteriocin | 19525 | Tetronasin | 2 | BGC0000163_c1 | | 3 |
| b62 | SDR | Butyrolactone | 19444 | gamma-butyrolactone | 3 | BGC0000849_c1 | | 100 |
| b62 | SDR | Butyrolactone-Ectoine | 15215 | Skyllamycin | 4 | BGC0000429_c1 | | 8 |
| b62 | SDR | Butyrolactone-Other PKS | 45560 | SF2575 | 5 | BGC0000269_c1 | | 6 |
| b62 | SDR | Ectoine | 7418 | Ectoine | 6 | BGC0000853_c1 | | 100 |
| b62 | SDR | Lantipeptide | 23314 | SRO15-3108 | 7 | BGC0000554_c1 | | 100 |
| b62 | SDR | Lantipeptide | 15770 | AmfS | 8 | BGC0000496_c1 | | 80 |
| b62 | SDR | Lassopeptide | 22703 | SRO15-2005 | 9 | BGC0000578_c1 | | 80 |
| b62 | SDR | Melanin | 8825 | Melanin | 10 | BGC0000911_c1 | | 100 |
| b62 | SDR | NRPS | 9430 | NA | 11 | NA | | NA |
| b62 | SDR | NRPS | 27486 | Coelichelin | 12 | BGC0000325_c1 | | 72 |
| b62 | SDR | NRPS | 36081 | Griseobactin | 13 | BGC0000368_c1 | | 70 |
| b62 | SDR | NRPS | 64508 | Phosphonoglycans | 14 | BGC0000806_c1 | | 3 |
| b62 | SDR | NRPS-T1PKS | 35029 | Enduracidin | 15 | BGC0000341_c1 | | 8 |
| b62 | SDR | NRPS-T1PKS | 39461 | SGR PTMs | 16 | BGC0001043_c1 | | 100 |
| b62 | SDR | NRPS-T1PKS | 67449 | C-1027 | 17 | BGC0000965_c1 | | 3 |
| b62 | SDR | Siderophore | 11778 | Desferrioxamine B | 18 | BGC0000941_c1 | | 100 |
| b62 | SDR | Siderophore | 14732 | NA | 19 | NA | | NA |
| b62 | SDR | T2PKS-T1PKS-Other PKS | 56429 | Hedamycin | 20 | BGC0000233_c1 | | 87 |
| b62 | SDR | T3PKS | 41118 | Herboxidiene | 21 | BGC0001065_c1 | | 6 |
| b62 | SDR | T3PKS | 41052 | Alkylresorcinol | 22 | BGC0000282_c1 | | 100 |
| b62 | SDR | Terpene | 21214 | Steffimycin | 23 | BGC0000273_c1 | | 19 |
| b62 | SDR | Terpene | 23534 | Isorenieratene | 24 | BGC0000664_c1 | | 85 |
| b62 | SDR | Terpene | 6042 | NA | 25 | NA | | NA |
| b62 | SDR | Terpene | 12647 | 2-methylisoborneol | 26 | BGC0000658_c1 | | 100 |
| b62 | SDR | Terpene | 25601 | Isorenieratene | 27 | BGC0000664_c1 | | 85 |
| b62 | SDR | Terpene | 22213 | NA | 28 | NA | | NA |
| b62 | SDR | Terpene | 21049 | Alnumycin | 29 | BGC0000195_c1 | | 9 |
| b62 | SDR | Terpene | 16702 | Hopene | 30 | BGC0000663_c1 | | 30 |
| b84 | NDR | Bacteriocin | 11328 | NA | 1 | NA | | NA |
| b84 | NDR | Butyrolactone | 10944 | Oxazolomycin | 3 | BGC0001106_c1 | | 6 |
| b84 | NDR | Ectoine | 10398 | Ectoine | 6 | BGC0000853_c1 | | 75 |
| b84 | NDR | Lantipeptide | 16071 | AmfS | 8 | BGC0000496_c1 | | 80 |
| b84 | NDR | Melanin | 10479 | Melanin | 10 | BGC0000911_c1 | | 100 |
| b84 | NDR | NRPS | 37842 | Coelichelin | 12 | BGC0000325_c1 | | 90 |
| b84 | NDR | NRPS | 47685 | Griseobactin | 13 | BGC0000368_c1 | | 94 |
| b84 | NDR | T1PKS | 26556 | NA | 15 | NA | | NA |
| b84 | NDR | NRPS-T1PKS | 49497 | SGR PTMs | 16 | BGC0001043_c1 | | 83 |
| b84 | NDR | Siderophore | 30849 | NA | 19 | NA | | NA |
| b84 | NDR | T3PKS | 35955 | Herboxidiene | 21 | BGC0001065_c1 | | 6 |
| b84 | NDR | Terpene | 13748 | Steffimycin | 23 | BGC0000273_c1 | | 11 |
| b84 | NDR | Terpene | 33766 | A54145 | 28 | BGC0000291_c1 | | 6 |
| b84 | NDR | Terpene | 23785 | Hopene | 30 | BGC0000663_c1 | | 61 |
| b84 | NDR | Arylpolyene-Ladderane-NRPS | 88443 | Skyllamycin | 31 | BGC0000429_c1 | | 38 |
| b84 | NDR | Bacteriocin | 7405 | NA | 32 | NA | | NA |
| b84 | NDR | Butyrolactone | 11986 | Lactonamycin | 33 | BGC0000238_c1 | | 3 |
| b84 | NDR | Ectoine | 5852 | Ectoine | 34 | BGC0000853_c1 | | 50 |
| b84 | NDR | Lantipeptide | 23305 | NA | 35 | NA | | NA |
| b84 | NDR | Lassopeptide | 24063 | NA | 36 | NA | | NA |
| b84 | NDR | Lassopeptide | 22726 | NA | 37 | NA | | NA |
| b84 | NDR | Melanin | 10422 | Melanin | 38 | BGC0000912_c1 | | 100 |
| b84 | NDR | NRPS | 26884 | Tetrocarcin A | 39 | BGC0000162_c1 | | 4 |
| b84 | NDR | NRPS | 12605 | Enduracidin | 40 | BGC0000341_c1 | | 10 |
| b84 | NDR | NRPS | 31012 | NA | 41 | NA | | NA |
| b84 | NDR | NRPS | 28133 | NA | 42 | NA | | NA |
| b84 | NDR | NRPS | 49711 | Arylomycin | 43 | BGC0000306_c1 | | 22 |
| b84 | NDR | NRPS-T1PKS | 33629 | Kanamycin | 44 | BGC0000703_c1 | | 2 |
| b84 | NDR | NRPS-T1PKS-T3PKS-Ectoine | 137666 | Lobophorin | 45 | BGC0001183_c1 | | 10 |
| b84 | NDR | Other | 35025 | Cycloheximide/actiphenol | 46 | BGC0000175_c1 | | 33 |
| b84 | NDR | Other PKS | 40995 | Lactonamycin | 47 | BGC0000238_c1 | | 15 |
| b84 | NDR | Siderophore | 7643 | Desferrioxamine B | 48 | BGC0000941_c1 | | 100 |
| b84 | NDR | T1PKS | 2455 | NA | 49 | NA | | NA |
| b84 | NDR | T2PKS | 42497 | Kiamycin | 50 | BGC0000235_c1 | | 95 |
| b84 | NDR | T2PKS | 34211 | Nonactin | 51 | BGC0000252_c1 | | 85 |
| b84 | NDR | T3PKS | 17754 | Alkylresorcinol | 52 | BGC0000282_c1 | | 100 |
| b84 | NDR | T3PKS | 20732 | Galbonolides | 53 | BGC0000065_c1 | | 6 |
| b84 | NDR | Terpene | 21313 | 2-methylisoborneol | 54 | BGC0000658_c1 | | 100 |
| b84 | NDR | Terpene | 17309 | NA | 55 | NA | | NA |
| b84 | NDR | Thiopeptide | 26233 | Rabelomycin | 56 | BGC0000262_c1 | | 10 |
| b94 | INT | Ectoine-Butyrolactone | 22594 | Skyllamycin | 4 | BGC0000429_c1 | | 12 |
| b94 | INT | Ectoine | 17093 | Ectoine | 6 | BGC0000853_c1 | | 75 |
| b94 | INT | Lassopeptide | 12912 | SRO15-2005 | 9 | BGC0000578_c1 | | 60 |
| b94 | INT | Melanin | 10270 | Melanin | 10 | BGC0000911_c1 | | 100 |
| b94 | INT | NRPS | 24882 | Coelichelin | 12 | BGC0000325_c1 | | 72 |
| b94 | INT | T1PKS | 14442 | NA | 15 | NA | | NA |
| b94 | INT | Siderophore | 26746 | Desferrioxamine B | 18 | BGC0000941_c1 | | 100 |
| b94 | INT | Terpene | 13661 | Steffimycin | 23 | BGC0000273_c1 | | 11 |
| b94 | INT | Terpene | 22234 | NA | 28 | NA | | NA |
| b94 | INT | Terpene | 13000 | Hopene | 30 | BGC0000663_c1 | | 30 |
| b94 | INT | Bacteriocin | 4115 | NA | 57 | NA | | NA |
| b94 | INT | Butyrolactone | 4304 | NA | 58 | NA | | NA |
| b94 | INT | Ladderane-Arylpolyene | 28669 | Skyllamycin | 59 | BGC0000429_c1 | | 20 |
| b94 | INT | Lantipeptide | 37157 | NA | 60 | NA | | NA |
| b94 | INT | Lantipeptide | 6795 | NA | 61 | NA | | NA |
| b94 | INT | Melanin | 8049 | NA | 62 | NA | | NA |
| b94 | INT | NRPS | 11524 | NA | 63 | NA | | NA |
| b94 | INT | NRPS | 3806 | NA | 63 | NA | | NA |
| b94 | INT | NRPS | 12870 | Viomycin | 64 | BGC0000458_c1 | | 42 |
| b94 | INT | NRPS | 1802 | NA | 65 | NA | | NA |
| b94 | INT | NRPS | 17047 | NA | 66 | NA | | NA |
| b94 | INT | NRPS | 30324 | Lankamycin | 67 | BGC0000085_c1 | | 16 |
| b94 | INT | NRPS | 29135 | Viomycin | 68 | BGC0000458_c1 | | 28 |
| b94 | INT | NRPS | 16214 | NA | 69 | NA | | NA |
| b94 | INT | NRPS-T1PKS | 19840 | SGR PTMs | 70 | BGC0001043_c1 | | 100 |
| b94 | INT | Other | 12653 | Echosides | 71 | BGC0000340_c1 | | 17 |
| b94 | INT | Other | 43526 | NA | 72 | NA | | NA |
| b94 | INT | Other | 10411 | Rubradirin | 73 | BGC0000141_c1 | | 3 |
| b94 | INT | Other | 8632 | NA | 74 | NA | | NA |
| b94 | INT | Phosphonate | 22893 | Rhizocticin | 75 | BGC0000926_c1 | | 6 |
| b94 | INT | Siderophore | 10118 | NA | 76 | NA | | NA |
| b94 | INT | Siderophore | 14037 | Kinamycin | 77 | BGC0000236_c1 | | 8 |
| b94 | INT | T2PKS | 24011 | Griseorhodin | 78 | BGC0000230_c1 | | 39 |
| b94 | INT | T3PKS | 2019 | NA | 79 | NA | | NA |
| b94 | INT | T3PKS | 11948 | Herboxidiene | 80 | BGC0001065_c1 | | 6 |
| b94 | INT | Terpene | 3233 | NA | 81 | NA | | NA |
| b94 | INT | Thiopeptide | 11186 | NA | 82 | NA | | NA |
| b94 | INT | Linaridin | 6194 | NA | NA | NA | | NA |
| b94 | INT | Other | 1952 | NA | NA | NA | | NA |
| b94 | INT | Other | 5598 | NA | NA | NA | | NA |
| b94 | INT | Other | 1927 | NA | NA | NA | | NA |
| b94 | INT | Other | 1903 | NA | NA | NA | | NA |
| b94 | INT | T1PKS | 5929 | NA | NA | NA | | NA |
| f150 | SDR | Bacteriocin | 11382 | NA | 1 | NA | | NA |
| f150 | SDR | Butyrolactone | 10905 | gamma-butyrolactone | 3 | BGC0000849_c1 | | 100 |
| f150 | SDR | Ectoine-Butyrolactone | 15277 | Pristinamycin | 4 | BGC0000952 c2 | | 17 |
| f150 | SDR | Butyrolactone-Other PKS | 46776 | SF2575 | 5 | BGC0000269_c1 | | 6 |
| f150 | SDR | Ectoine | 20197 | Ectoine | 6 | BGC0000853_c1 | | 75 |
| f150 | SDR | Lantipeptide | 23314 | SRO15-3108 | 7 | BGC0000554_c1 | | 100 |
| f150 | SDR | Lantipeptide | 22741 | AmfS | 8 | BGC0000496_c1 | | 100 |
| f150 | SDR | Lassopeptide | 22697 | SRO15-2005 | 9 | BGC0000578_c1 | | 80 |
| f150 | SDR | Melanin | 26096 | Istamycin | 10 | BGC0000700_c1 | | 4 |
| f150 | SDR | NRPS | 9389 | NA | 11 | NA | | NA |
| f150 | SDR | NRPS | 36450 | Coelichelin | 12 | BGC0000325_c1 | | 72 |
| f150 | SDR | NRPS | 56787 | Griseobactin | 13 | BGC0000368_c1 | | 100 |
| f150 | SDR | NRPS-T1PKS | 38985 | Leinamycin | 15 | BGC0001101_c1 | | 4 |
| f150 | SDR | NRPS-T1PKS | 49398 | SGR PTMs | 16 | BGC0001043_c1 | | 100 |
| f150 | SDR | T1PKS-NRPS | 57364 | C-1027 | 17 | BGC0000965_c1 | | 3 |
| f150 | SDR | Siderophore | 11778 | Desferrioxamine B | 18 | BGC0000941_c1 | | 80 |
| f150 | SDR | Siderophore | 14732 | NA | 19 | NA | | NA |
| f150 | SDR | T2PKS-T1PKS-Other PKS | 54071 | Hedamycin | 20 | BGC0000233_c1 | | 87 |
| f150 | SDR | T3PKS | 41118 | Herboxidiene | 21 | BGC0001065_c1 | | 6 |
| f150 | SDR | Terpene | 24345 | Steffimycin | 23 | BGC0000273_c1 | | 19 |
| f150 | SDR | Terpene | 6110 | NA | 25 | NA | | NA |
| f150 | SDR | Terpene | 12648 | 2-methylisoborneol | 26 | BGC0000658_c1 | | 100 |
| f150 | SDR | Terpene | 37024 | Stenothricin | 28 | BGC0000431_c1 | | 13 |
| f150 | SDR | Terpene | 22187 | Hopene | 30 | BGC0000663_c1 | | 69 |
| f150 | SDR | Lantipeptide | 23044 | NA | 35 | NA | | NA |
| f150 | SDR | T3PKS | 21951 | Alkylresorcinol | 52 | BGC0000282_c1 | | 100 |
| f150 | SDR | Bacteriocin | 10800 | NA | 83 | NA | | NA |
| f150 | SDR | NRPS | 26846 | Griseoviridin | 84 | BGC0001097_c1 | | 15 |
| f150 | SDR | NRPS-T1PKS | 43253 | Collismycin A | 85 | BGC0000973_c1 | | 85 |
| f150 | SDR | NRPS | 46293 | Kanamycin | 86 | BGC0000703_c1 | | 3 |
| f150 | SDR | NRPS | 64972 | Tetronasin | 87 | BGC0000163_c1 | | 11 |
| f150 | SDR | Terpene | 25570 | Isorenieratene | 88 | BGC0000664_c1 | | 100 |
| f51 | SDR | Butyrolactone | 10887 | gamma-butyrolactone | 3 | BGC0000849_c1 | | 100 |
| f51 | SDR | Butyrolactone-Ectoine | 21813 | Pristinamycin | 4 | BGC0000952 c2 | | 17 |
| f51 | SDR | Lantipeptide | 12300 | AmfS | 8 | BGC0000496_c1 | | 100 |
| f51 | SDR | Melanin | 14714 | Melanin | 10 | BGC0000911_c1 | | 100 |
| f51 | SDR | NRPS | 28947 | Coelichelin | 12 | BGC0000325_c1 | | 81 |
| f51 | SDR | T1PKS | 23309 | Avilamycin A | 15 | BGC0000026_c1 | | 5 |
| f51 | SDR | Siderophore | 11778 | Desferrioxamine B | 18 | BGC0000941_c1 | | 80 |
| f51 | SDR | T1PKS-T2PKS | 34209 | Hedamycin | 20 | BGC0000233_c1 | | 59 |
| f51 | SDR | T3PKS | 35918 | Herboxidiene | 21 | BGC0001065_c1 | | 6 |
| f51 | SDR | Terpene | 11439 | Steffimycin | 23 | BGC0000273_c1 | | 11 |
| f51 | SDR | Terpene | 13652 | NA | 28 | NA | | NA |
| f51 | SDR | Terpene | 13639 | Hopene | 30 | BGC0000663_c1 | | 15 |
| f51 | SDR | Bacteriocin | 7479 | NA | 32 | NA | | NA |
| f51 | SDR | T3PKS | 3418 | NA | 79 | NA | | NA |
| f51 | SDR | Other | 15762 | Erythrochelin | 84 | BGC0000349_c1 | | 28 |
| f51 | SDR | Terpene | 18651 | Isorenieratene | 88 | BGC0000664_c1 | | 85 |
| f51 | SDR | Ectoine | 5034 | Ectoine | 89 | BGC0000853_c1 | | 75 |
| f51 | SDR | Lantipeptide | 17778 | SRO15-3108 | 90 | BGC0000554_c1 | | 75 |
| f51 | SDR | NRPS | 25072 | NA | 91 | NA | | NA |
| f51 | SDR | NRPS-T1PKS | 32568 | SGR PTMs | 92 | BGC0001043_c1 | | 83 |
| f51 | SDR | NRPS | 64637 | Laspartomycin | 93 | BGC0000379_c1 | | 6 |
| f51 | SDR | NRPS | 29947 | NA | 94 | NA | | NA |
| f51 | SDR | NRPS | 7341 | Griseobactin | 95 | BGC0000368_c1 | | 11 |
| f51 | SDR | NRPS-T1PKS | 33036 | Kanamycin | 96 | BGC0000703_c1 | | 2 |
| f51 | SDR | NRPS | 48228 | NA | 97 | NA | | NA |
| f51 | SDR | NRPS | 3146 | NA | 98 | NA | | NA |
| f51 | SDR | NRPS | 4195 | NA | 99 | NA | | NA |
| f51 | SDR | Other | 6870 | NA | 100 | NA | | NA |
| f51 | SDR | Other PKS | 8097 | Hedamycin | 101 | BGC0000233_c1 | | 18 |
| f51 | SDR | Phenazine | 31591 | Phenazine | 102 | BGC0001080_c1 | | 38 |
| f51 | SDR | Siderophore | 9367 | NA | 103 | NA | | NA |
| f51 | SDR | T1PKS | 13871 | Chalcomycin | 104 | BGC0000035_c1 | | 4 |
| f51 | SDR | Terpene | 12017 | 2-methylisoborneol | 105 | BGC0000658_c1 | | 100 |
| f51 | SDR | Terpene | 6016 | NA | 106 | NA | | NA |
| f51 | SDR | Terpene | 3097 | NA | 107 | NA | | NA |
| f51 | SDR | Other | 1811 | NA | NA | NA | | NA |
| gb14 | SDR | Bacteriocin | 10331 | NA | 1 | NA | | NA |
| gb14 | SDR | Butyrolactone | 10887 | gamma-butyrolactone | 3 | BGC0000849_c1 | | 100 |
| gb14 | SDR | Ectoine-Butyrolactone | 15220 | Pristinamycin | 4 | BGC0000952 c2 | | 17 |
| gb14 | SDR | Ectoine | 10398 | Ectoine | 6 | BGC0000853_c1 | | 100 |
| gb14 | SDR | Lantipeptide | 23314 | SRO15-3108 | 7 | BGC0000554_c1 | | 75 |
| gb14 | SDR | Lantipeptide | 22747 | AmfS | 8 | BGC0000496_c1 | | 80 |
| gb14 | SDR | Lassopeptide | 22703 | SRO15-2005 | 9 | BGC0000578_c1 | | 80 |
| gb14 | SDR | Melanin | 10479 | Melanin | 10 | BGC0000911_c1 | | 100 |
| gb14 | SDR | NRPS | 36213 | Coelichelin | 12 | BGC0000325_c1 | | 72 |
| gb14 | SDR | NRPS | 29118 | Griseobactin | 13 | BGC0000368_c1 | | 41 |
| gb14 | SDR | NRPS | 65348 | Jagaricin | 14 | BGC0001127_c1 | | 13 |
| gb14 | SDR | T1PKS-NRPS | 39058 | Enduracidin | 15 | BGC0000341_c1 | | 8 |
| gb14 | SDR | NRPS-T1PKS | 49422 | SGR PTMs | 16 | BGC0001043_c1 | | 100 |
| gb14 | SDR | NRPS-T1PKS | 68099 | C-1027 | 17 | BGC0000965_c1 | | 3 |
| gb14 | SDR | Siderophore | 11778 | Desferrioxamine B | 18 | BGC0000941_c1 | | 80 |
| gb14 | SDR | Siderophore | 14792 | NA | 19 | NA | | NA |
| gb14 | SDR | T2PKS-T1PKS-Other PKS | 56471 | Hedamycin | 20 | BGC0000233_c1 | | 84 |
| gb14 | SDR | T3PKS | 41118 | Herboxidiene | 21 | BGC0001065_c1 | | 6 |
| gb14 | SDR | Terpene | 21076 | Steffimycin | 23 | BGC0000273_c1 | | 19 |
| gb14 | SDR | Terpene | 36848 | Stenothricin | 28 | BGC0000431_c1 | | 13 |
| gb14 | SDR | Terpene | 20989 | Alnumycin | 29 | BGC0000195_c1 | | 9 |
| gb14 | SDR | Terpene | 22148 | Hopene | 30 | BGC0000663_c1 | | 69 |
| gb14 | SDR | Lantipeptide | 27649 | NA | 35 | NA | | NA |
| gb14 | SDR | Trans-AT PKS-NRPS | 59848 | Fostriecin | 54 | BGC0000060_c1 | | 9 |
| gb14 | SDR | Terpene | 13425 | NA | 55 | NA | | NA |
| gb14 | SDR | T3PKS | 3436 | NA | 79 | NA | | NA |
| gb14 | SDR | Bacteriocin | 10800 | NA | 83 | NA | | NA |
| gb14 | SDR | NRPS | 27390 | Griseoviridin | 84 | BGC0001097_c1 | | 15 |
| gb14 | SDR | NRPS | 68554 | Tetronasin | 87 | BGC0000163_c1 | | 11 |
| gb14 | SDR | NRPS-T1PKS | 52260 | Daptomycin | 96 | BGC0000336_c1 | | 7 |
| gb14 | SDR | Phenazine | 20488 | Phenazine | 102 | BGC0001080_c1 | | 38 |
| gb14 | SDR | Lantipeptide | 22912 | NA | 108 | NA | | NA |
| gb14 | SDR | NRPS-T1PKS-Trans-AT PKS | 52736 | Xenortide | 109 | BGC0000465_c1 | | 100 |
| gb14 | SDR | Terpene | 20440 | 2-methylisoborneol | 110 | BGC0000658_c1 | | 100 |
| gb14 | SDR | Terpene | 25570 | Isorenieratene | 111 | BGC0000664_c1 | | 100 |
| gb14 | SDR | NRPS | 2123 | NA | NA | NA | | NA |
| man185 | INT | Bacteriocin | 11331 | NA | 1 | NA | | NA |
| man185 | INT | Butyrolactone | 10850 | gamma-butyrolactone | 3 | BGC0000849_c1 | | 100 |
| man185 | INT | Ectoine-Butyrolactone | 15348 | Pristinamycin | 4 | BGC0000952 c2 | | 17 |
| man185 | INT | Ectoine | 10398 | Ectoine | 6 | BGC0000853_c1 | | 75 |
| man185 | INT | Lantipeptide | 18092 | AmfS | 8 | BGC0000496_c1 | | 80 |
| man185 | INT | Lassopeptide | 22655 | SRO15-2005 | 9 | BGC0000578_c1 | | 80 |
| man185 | INT | Melanin | 10473 | Melanin | 10 | BGC0000911_c1 | | 100 |
| man185 | INT | NRPS | 34522 | Coelichelin | 12 | BGC0000325_c1 | | 72 |
| man185 | INT | NRPS | 51622 | Griseobactin | 13 | BGC0000368_c1 | | 100 |
| man185 | INT | T1PKS | 26561 | NA | 15 | NA | | NA |
| man185 | INT | NRPS-T1PKS | 36265 | SGR PTMs | 16 | BGC0001043_c1 | | 100 |
| man185 | INT | Siderophore | 11778 | Desferrioxamine B | 18 | BGC0000941_c1 | | 80 |
| man185 | INT | Siderophore | 14711 | NA | 19 | NA | | NA |
| man185 | INT | T3PKS | 41118 | Herboxidiene | 21 | BGC0001065_c1 | | 5 |
| man185 | INT | T3PKS | 41052 | Tetronasin | 22 | BGC0000163_c1 | | 11 |
| man185 | INT | Terpene | 21076 | Steffimycin | 23 | BGC0000273_c1 | | 19 |
| man185 | INT | Terpene | 22213 | NA | 28 | NA | | NA |
| man185 | INT | Terpene | 26573 | Hopene | 30 | BGC0000663_c1 | | 69 |
| man185 | INT | Lantipeptide | 32442 | NA | 35 | NA | | NA |
| man185 | INT | Terpene | 20977 | NA | 55 | NA | | NA |
| man185 | INT | NRPS-T1PKS | 33144 | Kanamycin | 96 | BGC0000703_c1 | | 1 |
| man185 | INT | Other | 6863 | NA | 100 | NA | | NA |
| man185 | INT | Lantipeptide | 22909 | NA | 108 | NA | | NA |
| man185 | INT | Bacteriocin | 14210 | NA | 112 | NA | | NA |
| man185 | INT | Lantipeptide-Arylpolyene | 30739 | Colabomycin | 113 | BGC0000213_c1 | | 11 |
| man185 | INT | NRPS | 43867 | Concanamycin A | 114 | BGC0000040_c1 | | 21 |
| man185 | INT | NRPS | 51201 | Azicemicin | 115 | BGC0000202_c1 | | 11 |
| man185 | INT | Other PKS-T1PKS | 49564 | NA | 116 | NA | | NA |
| man185 | INT | T1PKS | 20243 | Stambomycin | 117 | BGC0000151_c1 | | 56 |
| man185 | INT | T1PKS | 55445 | Neocarzilin | 118 | BGC0000111_c1 | | 57 |
| man185 | INT | Terpene | 17048 | Isorenieratene | 119 | BGC0000664_c1 | | 100 |
| man185 | INT | Thiopeptide-Lantipeptide | 26414 | NA | 120 | NA | | NA |
| man185 | INT | Trans-AT PKS | 62178 | Sorangicin | 121 | BGC0000184_c1 | | 8 |
| man185 | INT | Trans-AT PKS-Other PKS | 46498 | Calyculin | 122 | BGC0000967_c1 | | 32 |
| man185 | INT | Trans-AT PKS | 4739 | NA | NA | NA | | NA |
| man185 | INT | T1PKS | 9892 | Sanglifehrin A | NA | BGC0001042_c1 | | 11 |
| man185 | INT | T1PKS | 15672 | Nystatin | NA | BGC0000115_c1 | | 27 |
| man185 | INT | T1PKS | 5018 | Fostriecin | NA | BGC0000060_c1 | | 28 |
| ms115 | SDR | Bacteriocin | 11382 | NA | 1 | NA | | NA |
| ms115 | SDR | Butyrolactone | 10944 | gamma-butyrolactone | 3 | BGC0000849_c1 | | 100 |
| ms115 | SDR | Butyrolactone-Ectoine | 15216 | Pristinamycin | 4 | BGC0000952 c2 | | 17 |
| ms115 | SDR | Other PKS-Butyrolactone | 61034 | SF2575 | 5 | BGC0000269_c1 | | 6 |
| ms115 | SDR | Ectoine | 10398 | Ectoine | 6 | BGC0000853_c1 | | 100 |
| ms115 | SDR | Lantipeptide | 23314 | SRO15-3108 | 7 | BGC0000554_c1 | | 75 |
| ms115 | SDR | Lantipeptide | 18108 | AmfS | 8 | BGC0000496_c1 | | 100 |
| ms115 | SDR | Lassopeptide | 23115 | SRO15-2005 | 9 | BGC0000578_c1 | | 80 |
| ms115 | SDR | Melanin | 10479 | Istamycin | 10 | BGC0000700_c1 | | 4 |
| ms115 | SDR | NRPS | 36629 | Coelichelin | 12 | BGC0000325_c1 | | 81 |
| ms115 | SDR | NRPS | 56812 | Griseobactin | 13 | BGC0000368_c1 | | 100 |
| ms115 | SDR | NRPS | 65354 | Phosphonoglycans | 14 | BGC0000806_c1 | | 3 |
| ms115 | SDR | T1PKS-NRPS | 38925 | Enduracidin | 15 | BGC0000341_c1 | | 6 |
| ms115 | SDR | NRPS-T1PKS | 49344 | SGR PTMs | 16 | BGC0001043_c1 | | 83 |
| ms115 | SDR | T1PKS-NRPS | 68052 | C-1027 | 17 | BGC0000965_c1 | | 3 |
| ms115 | SDR | Siderophore | 11778 | Desferrioxamine B | 18 | BGC0000941_c1 | | 80 |
| ms115 | SDR | Siderophore | 14768 | NA | 19 | NA | | NA |
| ms115 | SDR | T2PKS-T1PKS-Other PKS | 54107 | Hedamycin | 20 | BGC0000233_c1 | | 87 |
| ms115 | SDR | T3PKS | 41118 | Herboxidiene | 21 | BGC0001065_c1 | | 6 |
| ms115 | SDR | T3PKS | 41052 | Tetronasin | 22 | BGC0000163_c1 | | 11 |
| ms115 | SDR | Terpene | 18757 | Steffimycin | 23 | BGC0000273_c1 | | 19 |
| ms115 | SDR | Terpene | 32731 | NA | 28 | NA | | NA |
| ms115 | SDR | Terpene | 22187 | Hopene | 30 | BGC0000663_c1 | | 69 |
| ms115 | SDR | Terpene | 20866 | NA | 55 | NA | | NA |
| ms115 | SDR | Bacteriocin | 10355 | NA | 83 | NA | | NA |
| ms115 | SDR | NRPS-T1PKS | 32999 | Kanamycin | 96 | BGC0000703_c1 | | 2 |
| ms115 | SDR | Lantipeptide | 17366 | NA | 123 | NA | | NA |
| ms115 | SDR | Terpene-NRPS | 41451 | Isorenieratene | 124 | BGC0000664_c1 | | 100 |
| ms184 | SDR | Butyrolactone | 11958 | gamma-butyrolactone | 3 | BGC0000849_c1 | | 100 |
| ms184 | SDR | Lassopeptide | 17994 | SRO15-2005 | 9 | BGC0000578_c1 | | 60 |
| ms184 | SDR | Melanin | 7477 | Istamycin | 10 | BGC0000700_c1 | | 4 |
| ms184 | SDR | T1PKS | 13265 | NA | 15 | NA | | NA |
| ms184 | SDR | Siderophore | 11778 | Desferrioxamine B | 18 | BGC0000941_c1 | | 100 |
| ms184 | SDR | Siderophore | 17237 | NA | 19 | NA | | NA |
| ms184 | SDR | T3PKS | 22822 | Herboxidiene | 21 | BGC0001065_c1 | | 5 |
| ms184 | SDR | Terpene | 11343 | Steffimycin | 23 | BGC0000273_c1 | | 11 |
| ms184 | SDR | Terpene | 9512 | Hopene | 30 | BGC0000663_c1 | | 15 |
| ms184 | SDR | Bacteriocin | 7464 | NA | 32 | NA | | NA |
| ms184 | SDR | T3PKS | 4591 | Alkylresorcinol | 79 | BGC0000282_c1 | | 66 |
| ms184 | SDR | Terpene | 3007 | NA | 81 | NA | | NA |
| ms184 | SDR | Thiopeptide | 16857 | NA | 82 | NA | | NA |
| ms184 | SDR | Ectoine | 4973 | Ectoine | 89 | BGC0000853_c1 | | 100 |
| ms184 | SDR | NRPS-T1PKS | 32206 | SGR PTMs | 92 | BGC0001043_c1 | | 100 |
| ms184 | SDR | NRPS | 5070 | Griseobactin | 95 | BGC0000368_c1 | | 11 |
| ms184 | SDR | Terpene | 3428 | NA | 107 | NA | | NA |
| ms184 | SDR | Butyrolactone | 17521 | Pristinamycin | 125 | BGC0000952 c3 | | 2 |
| ms184 | SDR | Lantipeptide | 4194 | AmfS | 126 | BGC0000496_c1 | | 40 |
| ms184 | SDR | NRPS | 10263 | Lobophorin | 127 | BGC0001183_c1 | | 3 |
| ms184 | SDR | NRPS | 18776 | Coelichelin | 128 | BGC0000325_c1 | | 27 |
| ms184 | SDR | NRPS | 6715 | A54145 | 129 | BGC0000291_c1 | | 8 |
| ms184 | SDR | NRPS | 26273 | Coelibactin | 130 | BGC0000324_c1 | | 36 |
| ms184 | SDR | NRPS | 23940 | Balhimycin | 131 | BGC0000311_c1 | | 5 |
| ms184 | SDR | NRPS | 5426 | NA | 132 | NA | | NA |
| ms184 | SDR | NRPS | 16205 | Enduracidin | 133 | BGC0000341_c1 | | 10 |
| ms184 | SDR | Other | 4798 | NA | 134 | NA | | NA |
| ms184 | SDR | Other | 31912 | Viguiepinol | 135 | BGC0000286_c1 | | 73 |
| ms184 | SDR | Other | 18450 | Daptomycin | 136 | BGC0000336_c1 | | 12 |
| ms184 | SDR | Phenazine-Other PKS | 9610 | Phenazine | 137 | BGC0001080_c1 | | 38 |
| ms184 | SDR | T1PKS | 10086 | Esmeraldin | 138 | BGC0000935_c1 | | 28 |
| ms184 | SDR | T2PKS | 16462 | Sch47554/Sch47555 | 139 | BGC0000268_c1 | | 14 |
| ms184 | SDR | Terpene | 13764 | Isorenieratene | 140 | BGC0000664_c1 | | 57 |
| ms184 | SDR | Terpene | 11849 | Isorenieratene | 141 | BGC0000664_c1 | | 42 |
| ms184 | SDR | Terpene | 19256 | NA | 142 | NA | | NA |
| ms184 | SDR | Terpene | 3917 | NA | 143 | NA | | NA |
| ms184 | SDR | NRPS | 3364 | NA | NA | NA | | NA |
| ms184 | SDR | NRPS | 2140 | NA | NA | NA | | NA |
| ms184 | SDR | NRPS | 8926 | NA | NA | NA | | NA |
| ms184 | SDR | NRPS | 3463 | NA | NA | NA | | NA |
| ms184 | SDR | NRPS | 1180 | NA | NA | NA | | NA |
| ms184 | SDR | NRPS | 10611 | NA | NA | NA | | NA |
| or20 | NDR | Bacteriocin | 11358 | NA | 1 | NA | | NA |
| or20 | NDR | Butyrolactone | 10875 | Oxazolomycin | 3 | BGC0001106_c1 | | 6 |
| or20 | NDR | Ectoine | 10398 | Ectoine | 6 | BGC0000853_c1 | | 100 |
| or20 | NDR | Lantipeptide | 15971 | AmfS | 8 | BGC0000496_c1 | | 80 |
| or20 | NDR | Melanin | 12028 | Melanin | 10 | BGC0000911_c1 | | 100 |
| or20 | NDR | NRPS | 36877 | Coelichelin | 12 | BGC0000325_c1 | | 90 |
| or20 | NDR | NRPS | 48561 | Griseobactin | 13 | BGC0000368_c1 | | 94 |
| or20 | NDR | T1PKS-NRPS | 32431 | Landomycin | 15 | BGC0000239_c1 | | 6 |
| or20 | NDR | NRPS-T1PKS | 39003 | SGR PTMs | 16 | BGC0001043_c1 | | 100 |
| or20 | NDR | Siderophore | 14710 | NA | 19 | NA | | NA |
| or20 | NDR | T3PKS | 41118 | Herboxidiene | 21 | BGC0001065_c1 | | 6 |
| or20 | NDR | Terpene | 13798 | Steffimycin | 23 | BGC0000273_c1 | | 11 |
| or20 | NDR | Terpene | 22213 | NA | 28 | NA | | NA |
| or20 | NDR | Terpene | 23781 | Hopene | 30 | BGC0000663_c1 | | 69 |
| or20 | NDR | Bacteriocin | 7536 | NA | 32 | NA | | NA |
| or20 | NDR | Lantipeptide | 23236 | NA | 35 | NA | | NA |
| or20 | NDR | NRPS | 26906 | Incednine | 39 | BGC0000078_c1 | | 2 |
| or20 | NDR | Other | 23825 | Arylomycin | 43 | BGC0000306_c1 | | 22 |
| or20 | NDR | Terpene | 23439 | 2-methylisoborneol | 54 | BGC0000658_c1 | | 100 |
| or20 | NDR | Terpene | 16823 | NA | 55 | NA | | NA |
| or20 | NDR | Thiopeptide | 26281 | Rabelomycin | 56 | BGC0000262_c1 | | 10 |
| or20 | NDR | Other PKS-T1PKS | 44177 | Concanamycin A | 116 | BGC0000040_c1 | | 21 |
| or20 | NDR | Butyrolactone | 8016 | Skyllamycin | 144 | BGC0000429_c1 | | 8 |
| or20 | NDR | Ectoine | 13229 | Pristinamycin | 145 | BGC0000952 c2 | | 23 |
| or20 | NDR | Ladderane-Arylpolyene-NRPS | 77093 | Skyllamycin | 146 | BGC0000429_c1 | | 73 |
| or20 | NDR | Lantipeptide | 28519 | Cycloheximide/actiphenol | 147 | BGC0000175_c1 | | 44 |
| or20 | NDR | Linaridin | 20608 | NA | 148 | NA | | NA |
| or20 | NDR | Melanin | 10365 | Grixazone | 149 | BGC0000662_c1 | | 76 |
| or20 | NDR | Melanin | 10422 | Melanin | 150 | BGC0000912_c1 | | 100 |
| or20 | NDR | NRPS | 51915 | Capreomycin | 151 | BGC0000316_c1 | | 12 |
| or20 | NDR | NRPS | 31901 | Tomaymycin | 152 | BGC0000448_c1 | | 35 |
| or20 | NDR | NRPS-T1PKS | 50881 | NA | 153 | NA | | NA |
| or20 | NDR | NRPS | 73718 | Daptomycin | 154 | BGC0000336_c1 | | 3 |
| or20 | NDR | NRPS | 9513 | NA | 155 | NA | | NA |
| or20 | NDR | NRPS | 39245 | Borrelidin | 156 | BGC0000031_c1 | | 9 |
| or20 | NDR | NRPS | 31959 | Amicetin | 157 | BGC0000953_c1 | | 8 |
| or20 | NDR | Oligosaccharide-T2PKS-NRPS | 78766 | Landomycin | 158 | BGC0000239_c1 | | 68 |
| or20 | NDR | Other | 14506 | NA | 159 | NA | | NA |
| or20 | NDR | Other | 22405 | NA | 160 | NA | | NA |
| or20 | NDR | Other PKS | 27895 | Calcium-dependent antibiotic | 161 | BGC0000315_c1 | | 7 |
| or20 | NDR | Siderophore | 11778 | Desferrioxamine B | 162 | BGC0000941_c1 | | 100 |
| or20 | NDR | T3PKS | 17141 | Alkylresorcinol | 163 | BGC0000282_c1 | | 66 |
| or20 | NDR | T3PKS | 34625 | NA | 164 | NA | | NA |
| or20 | NDR | Terpene | 18171 | Isorenieratene | 165 | BGC0000664_c1 | | 100 |
| or3 | NDR | Bacteriocin | 11328 | NA | 1 | NA | | NA |
| or3 | NDR | Butyrolactone | 10926 | Oxazolomycin | 3 | BGC0001106_c1 | | 6 |
| or3 | NDR | Butyrolactone-Ectoine | 15253 | Pristinamycin | 4 | BGC0000952 c2 | | 17 |
| or3 | NDR | Ectoine | 10398 | Ectoine | 6 | BGC0000853_c1 | | 100 |
| or3 | NDR | Lantipeptide | 21515 | AmfS | 8 | BGC0000496_c1 | | 100 |
| or3 | NDR | Melanin | 11960 | NA | 10 | NA | | NA |
| or3 | NDR | NRPS | 37010 | Coelichelin | 12 | BGC0000325_c1 | | 90 |
| or3 | NDR | NRPS | 46066 | Griseobactin | 13 | BGC0000368_c1 | | 94 |
| or3 | NDR | NRPS-T1PKS | 32465 | Enduracidin | 15 | BGC0000341_c1 | | 4 |
| or3 | NDR | NRPS-T1PKS | 43509 | SGR PTMs | 16 | BGC0001043_c1 | | 100 |
| or3 | NDR | Siderophore | 23104 | Kinamycin | 19 | BGC0000236_c1 | | 11 |
| or3 | NDR | T3PKS | 41088 | Herboxidiene | 21 | BGC0001065_c1 | | 6 |
| or3 | NDR | T3PKS | 41052 | Alkylresorcinol | 22 | BGC0000282_c1 | | 100 |
| or3 | NDR | Terpene | 21214 | Steffimycin | 23 | BGC0000273_c1 | | 19 |
| or3 | NDR | Terpene | 31124 | NA | 28 | NA | | NA |
| or3 | NDR | Terpene | 23766 | Hopene | 30 | BGC0000663_c1 | | 69 |
| or3 | NDR | Lantipeptide | 30556 | Cyclothiazomycin | 35 | BGC0000603_c1 | | 9 |
| or3 | NDR | NRPS | 27858 | Incednine | 39 | BGC0000078_c1 | | 4 |
| or3 | NDR | NRPS | 51778 | Daptomycin | 41 | BGC0000336_c1 | | 3 |
| or3 | NDR | NRPS | 38580 | Arylomycin | 43 | BGC0000306_c1 | | 22 |
| or3 | NDR | T2PKS | 33155 | Nonactin | 51 | BGC0000252_c1 | | 85 |
| or3 | NDR | Terpene | 25578 | 2-methylisoborneol | 54 | BGC0000658_c1 | | 100 |
| or3 | NDR | Terpene | 27048 | NA | 55 | NA | | NA |
| or3 | NDR | Thiopeptide | 26254 | Rabelomycin | 56 | BGC0000262_c1 | | 10 |
| or3 | NDR | Bacteriocin | 14634 | Tetronasin | 83 | BGC0000163_c1 | | 3 |
| or3 | NDR | Lantipeptide | 29403 | Cycloheximide/actiphenol | 147 | BGC0000175_c1 | | 44 |
| or3 | NDR | Linaridin | 16565 | NA | 148 | NA | | NA |
| or3 | NDR | Melanin | 19554 | Melanin | 150 | BGC0000912_c1 | | 100 |
| or3 | NDR | NRPS | 39034 | Fluostatin | 156 | BGC0000223_c1 | | 4 |
| or3 | NDR | NRPS-Oligosaccharide-T2PKS | 113767 | Landomycin | 158 | BGC0000239_c1 | | 71 |
| or3 | NDR | Other | 22413 | NA | 160 | NA | | NA |
| or3 | NDR | Siderophore | 11778 | Desferrioxamine B | 162 | BGC0000941_c1 | | 100 |
| or3 | NDR | Terpene | 22435 | Isorenieratene | 165 | BGC0000664_c1 | | 100 |
| or3 | NDR | Arylpolyene | 27902 | A201A | 166 | BGC0000873_c1 | | 12 |
| or3 | NDR | NRPS-Arylpolyene-Ladderane | 51202 | Skyllamycin | 167 | BGC0000429_c1 | | 38 |
| or3 | NDR | NRPS | 51491 | Calcium-dependent antibiotic | 168 | BGC0000315_c1 | | 10 |
| or3 | NDR | NRPS-T1PKS | 49734 | Skyllamycin | 169 | BGC0000429_c1 | | 22 |
| or3 | NDR | Other | 16267 | Tomaymycin | 170 | BGC0000448_c1 | | 17 |
| or3 | NDR | T1PKS | 45834 | C-1027 | 171 | BGC0000965_c1 | | 13 |
| or3 | NDR | T1PKS | 67305 | Tetronasin | 172 | BGC0000163_c1 | | 3 |
| rh195 | NDR | Bacteriocin | 11933 | NA | 1 | NA | | NA |
| rh195 | NDR | Butyrolactone | 10926 | Oxazolomycin | 3 | BGC0001106_c1 | | 6 |
| rh195 | NDR | Ectoine | 8042 | Ectoine | 6 | BGC0000853_c1 | | 75 |
| rh195 | NDR | Lantipeptide | 12903 | AmfS | 8 | BGC0000496_c1 | | 100 |
| rh195 | NDR | Melanin | 6525 | Istamycin | 10 | BGC0000700_c1 | | 4 |
| rh195 | NDR | NRPS | 32945 | Coelichelin | 12 | BGC0000325_c1 | | 90 |
| rh195 | NDR | T1PKS | 17139 | NA | 15 | NA | | NA |
| rh195 | NDR | NRPS-T1PKS | 33086 | SGR PTMs | 16 | BGC0001043_c1 | | 100 |
| rh195 | NDR | Siderophore | 19621 | Kinamycin | 19 | BGC0000236_c1 | | 5 |
| rh195 | NDR | T3PKS | 41088 | Herboxidiene | 21 | BGC0001065_c1 | | 4 |
| rh195 | NDR | Terpene | 21054 | NA | 28 | NA | | NA |
| rh195 | NDR | Terpene | 14102 | Hopene | 30 | BGC0000663_c1 | | 38 |
| rh195 | NDR | Bacteriocin | 7337 | NA | 32 | NA | | NA |
| rh195 | NDR | Siderophore | 7630 | Desferrioxamine B | 48 | BGC0000941_c1 | | 100 |
| rh195 | NDR | T3PKS | 17446 | Alkylresorcinol | 52 | BGC0000282_c1 | | 66 |
| rh195 | NDR | Terpene | 21310 | 2-methylisoborneol | 54 | BGC0000658_c1 | | 100 |
| rh195 | NDR | Other | 7558 | NA | 100 | NA | | NA |
| rh195 | NDR | Melanin | 8252 | Melanin | 150 | BGC0000912_c1 | | 100 |
| rh195 | NDR | Arylpolyene | 28018 | Hygromycin A | 166 | BGC0000698_c1 | | 13 |
| rh195 | NDR | Ladderane-Arylpolyene | 17158 | Skyllamycin | 167 | BGC0000429_c1 | | 30 |
| rh195 | NDR | T1PKS | 45858 | C-1027 | 171 | BGC0000965_c1 | | 13 |
| rh195 | NDR | Lantipeptide | 19643 | NA | 173 | NA | | NA |
| rh195 | NDR | Lantipeptide | 24647 | Kanamycin | 174 | BGC0000703_c1 | | 1 |
| rh195 | NDR | Linaridin | 6220 | NA | 175 | NA | | NA |
| rh195 | NDR | NRPS | 8949 | Enduracidin | 176 | BGC0000341_c1 | | 4 |
| rh195 | NDR | NRPS | 25861 | NA | 177 | NA | | NA |
| rh195 | NDR | NRPS | 7577 | NA | 178 | NA | | NA |
| rh195 | NDR | NRPS | 24566 | WAP-8294A2 (lotilibcin) | 179 | BGC0000461_c1 | | 30 |
| rh195 | NDR | NRPS | 64117 | Amicetin | 180 | BGC0000953_c1 | | 8 |
| rh195 | NDR | NRPS-T1PKS | 22734 | NA | 181 | NA | | NA |
| rh195 | NDR | NRPS | 21207 | NA | 182 | NA | | NA |
| rh195 | NDR | NRPS-T1PKS | 16853 | NA | 183 | NA | | NA |
| rh195 | NDR | Oligosaccharide-T2PKS | 37934 | Landomycin | 184 | BGC0000239_c1 | | 65 |
| rh195 | NDR | Other | 16785 | Cycloheximide/actiphenol | 185 | BGC0000175_c1 | | 22 |
| rh195 | NDR | Other PKS | 24014 | Cyclomarin | 186 | BGC0000333_c1 | | 8 |
| rh195 | NDR | Terpene | 5397 | NA | 187 | NA | | NA |
| rh195 | NDR | Terpene | 19500 | Isorenieratene | 188 | BGC0000664_c1 | | 85 |
| rh206 | NDR | Butyrolactone | 10421 | Oxazolomycin | 3 | BGC0001106_c1 | | 6 |
| rh206 | NDR | Ectoine | 8060 | Ectoine | 6 | BGC0000853_c1 | | 75 |
| rh206 | NDR | Lantipeptide | 13202 | AmfS | 8 | BGC0000496_c1 | | 100 |
| rh206 | NDR | Melanin | 8787 | Melanin | 10 | BGC0000911_c1 | | 100 |
| rh206 | NDR | NRPS | 8204 | NA | 11 | NA | | NA |
| rh206 | NDR | NRPS | 31607 | Coelichelin | 12 | BGC0000325_c1 | | 81 |
| rh206 | NDR | T1PKS | 17154 | NA | 15 | NA | | NA |
| rh206 | NDR | NRPS-T1PKS | 29051 | SGR PTMs | 16 | BGC0001043_c1 | | 100 |
| rh206 | NDR | Siderophore | 19673 | Kinamycin | 19 | BGC0000236_c1 | | 5 |
| rh206 | NDR | T3PKS | 27216 | Herboxidiene | 21 | BGC0001065_c1 | | 4 |
| rh206 | NDR | Terpene | 12965 | Steffimycin | 23 | BGC0000273_c1 | | 11 |
| rh206 | NDR | Terpene | 21078 | NA | 28 | NA | | NA |
| rh206 | NDR | Terpene | 13789 | Hopene | 30 | BGC0000663_c1 | | 38 |
| rh206 | NDR | Bacteriocin | 7410 | NA | 32 | NA | | NA |
| rh206 | NDR | Siderophore | 10914 | Desferrioxamine B | 48 | BGC0000941_c1 | | 100 |
| rh206 | NDR | Terpene | 23796 | 2-methylisoborneol | 54 | BGC0000658_c1 | | 100 |
| rh206 | NDR | Terpene | 14354 | NA | 55 | NA | | NA |
| rh206 | NDR | T3PKS | 2631 | NA | 79 | NA | | NA |
| rh206 | NDR | Other | 6877 | NA | 100 | NA | | NA |
| rh206 | NDR | Linaridin | 14092 | NA | 148 | NA | | NA |
| rh206 | NDR | Melanin | 11069 | Melanin | 150 | BGC0000912_c1 | | 100 |
| rh206 | NDR | Ladderane-Arylpolyene-NRPS | 51164 | Skyllamycin | 167 | BGC0000429_c1 | | 40 |
| rh206 | NDR | Lantipeptide | 19641 | NA | 173 | NA | | NA |
| rh206 | NDR | Lantipeptide | 24647 | Kanamycin | 174 | BGC0000703_c1 | | 1 |
| rh206 | NDR | NRPS | 24475 | Arylomycin | 179 | BGC0000306_c1 | | 22 |
| rh206 | NDR | NRPS | 59489 | Amicetin | 180 | BGC0000953_c1 | | 8 |
| rh206 | NDR | NRPS | 15573 | NA | 182 | NA | | NA |
| rh206 | NDR | Oligosaccharide-T2PKS | 37899 | Landomycin | 184 | BGC0000239_c1 | | 65 |
| rh206 | NDR | Other | 14777 | Cycloheximide/actiphenol | 185 | BGC0000175_c1 | | 16 |
| rh206 | NDR | Other PKS | 24069 | Cyclomarin | 186 | BGC0000333_c1 | | 8 |
| rh206 | NDR | Terpene | 18130 | Isorenieratene | 188 | BGC0000664_c1 | | 85 |
| rh206 | NDR | Arylpolyene | 14424 | A201A | 189 | BGC0000873_c1 | | 9 |
| rh206 | NDR | NRPS | 23533 | NA | 190 | NA | | NA |
| rh206 | NDR | NRPS | 3484 | NA | 191 | NA | | NA |
| rh207 | NDR | Bacteriocin | 9996 | NA | 1 | NA | | NA |
| rh207 | NDR | Butyrolactone | 15499 | Oxazolomycin | 3 | BGC0001106_c1 | | 6 |
| rh207 | NDR | Ectoine | 7968 | Ectoine | 6 | BGC0000853_c1 | | 75 |
| rh207 | NDR | Lantipeptide | 14648 | AmfS | 8 | BGC0000496_c1 | | 100 |
| rh207 | NDR | Melanin | 9568 | Istamycin | 10 | BGC0000700_c1 | | 4 |
| rh207 | NDR | NRPS | 27517 | Coelichelin | 12 | BGC0000325_c1 | | 90 |
| rh207 | NDR | T1PKS | 17290 | NA | 15 | NA | | NA |
| rh207 | NDR | NRPS-T1PKS | 38633 | SGR PTMs | 16 | BGC0001043_c1 | | 100 |
| rh207 | NDR | Siderophore | 23126 | Kinamycin | 19 | BGC0000236_c1 | | 11 |
| rh207 | NDR | T3PKS | 35998 | Herboxidiene | 21 | BGC0001065_c1 | | 4 |
| rh207 | NDR | Terpene | 15399 | NA | 28 | NA | | NA |
| rh207 | NDR | Terpene | 23725 | Hopene | 30 | BGC0000663_c1 | | 69 |
| rh207 | NDR | Bacteriocin | 11992 | NA | 32 | NA | | NA |
| rh207 | NDR | NRPS-T1PKS | 27486 | Balhimycin | 44 | BGC0000311_c1 | | 5 |
| rh207 | NDR | Terpene | 25453 | 2-methylisoborneol | 54 | BGC0000658_c1 | | 100 |
| rh207 | NDR | T3PKS | 2121 | NA | 79 | NA | | NA |
| rh207 | NDR | Other | 6923 | NA | 100 | NA | | NA |
| rh207 | NDR | Melanin | 8306 | Melanin | 150 | BGC0000912_c1 | | 100 |
| rh207 | NDR | Siderophore | 21067 | Desferrioxamine B | 162 | BGC0000941_c1 | | 100 |
| rh207 | NDR | Arylpolyene | 27976 | Hygromycin A | 166 | BGC0000698_c1 | | 13 |
| rh207 | NDR | Ladderane-Arylpolyene | 21784 | Skyllamycin | 167 | BGC0000429_c1 | | 26 |
| rh207 | NDR | T1PKS | 33594 | Neocarzinostatin | 171 | BGC0000112_c1 | | 15 |
| rh207 | NDR | Lantipeptide | 19641 | NA | 173 | NA | | NA |
| rh207 | NDR | Lantipeptide | 24647 | Kanamycin | 174 | BGC0000703_c1 | | 1 |
| rh207 | NDR | Linaridin | 4648 | NA | 175 | NA | | NA |
| rh207 | NDR | NRPS | 7630 | NA | 178 | NA | | NA |
| rh207 | NDR | NRPS | 24504 | WAP-8294A2 (lotilibcin) | 179 | BGC0000461_c1 | | 30 |
| rh207 | NDR | NRPS | 15998 | Fluostatin | 182 | BGC0000223_c1 | | 3 |
| rh207 | NDR | Oligosaccharide | 26639 | Landomycin | 184 | BGC0000239_c1 | | 34 |
| rh207 | NDR | Other | 18820 | Cycloheximide/actiphenol | 185 | BGC0000175_c1 | | 22 |
| rh207 | NDR | Other PKS | 24018 | Cyclomarin | 186 | BGC0000333_c1 | | 8 |
| rh207 | NDR | Terpene | 4898 | NA | 187 | NA | | NA |
| rh207 | NDR | Terpene | 18125 | Isorenieratene | 188 | BGC0000664_c1 | | 85 |
| rh207 | NDR | NRPS | 24944 | NA | 190 | NA | | NA |
| rh207 | NDR | NRPS | 3430 | NA | 191 | NA | | NA |
| rh207 | NDR | NRPS | 20389 | NA | 192 | NA | | NA |
| rh207 | NDR | NRPS | 16106 | NA | 193 | NA | | NA |
| rh207 | NDR | NRPS | 19847 | NA | 194 | NA | | NA |
| rh207 | NDR | T2PKS | 11222 | Simocyclinone | 195 | BGC0001072_c1 | | 18 |
| rh207 | NDR | Trans-AT PKS | 7778 | NA | NA | NA | | NA |
| rh34 | NDR | Ectoine | 20254 | Ectoine | 6 | BGC0000853_c1 | | 100 |
| rh34 | NDR | Lantipeptide | 12165 | AmfS | 8 | BGC0000496_c1 | | 100 |
| rh34 | NDR | Melanin | 9014 | Istamycin | 10 | BGC0000700_c1 | | 4 |
| rh34 | NDR | Siderophore | 23229 | Kinamycin | 19 | BGC0000236_c1 | | 11 |
| rh34 | NDR | T3PKS | 27854 | Herboxidiene | 21 | BGC0001065_c1 | | 6 |
| rh34 | NDR | Terpene | 13896 | Steffimycin | 23 | BGC0000273_c1 | | 11 |
| rh34 | NDR | Terpene | 26106 | NA | 28 | NA | | NA |
| rh34 | NDR | Terpene | 10435 | Phosphonoglycans | 30 | BGC0000807_c1 | | 6 |
| rh34 | NDR | Siderophore | 7639 | Desferrioxamine B | 48 | BGC0000941_c1 | | 80 |
| rh34 | NDR | NRPS | 7261 | Griseobactin | 95 | BGC0000368_c1 | | 11 |
| rh34 | NDR | NRPS-T1PKS | 33518 | Kanamycin | 96 | BGC0000703_c1 | | 2 |
| rh34 | NDR | Other | 6864 | NA | 100 | NA | | NA |
| rh34 | NDR | Melanin | 10422 | NA | 150 | NA | | NA |
| rh34 | NDR | Ladderane-Arylpolyene | 20148 | Skyllamycin | 167 | BGC0000429_c1 | | 24 |
| rh34 | NDR | Bacteriocin | 3676 | NA | 196 | NA | | NA |
| rh34 | NDR | Butyrolactone | 9764 | NA | 197 | NA | | NA |
| rh34 | NDR | Lantipeptide | 4907 | NA | 198 | NA | | NA |
| rh34 | NDR | Lantipeptide | 31035 | Novobiocin | 199 | BGC0000834_c1 | | 16 |
| rh34 | NDR | NRPS-T1PKS | 21668 | NA | 200 | NA | | NA |
| rh34 | NDR | NRPS | 42004 | Friulimicin | 201 | BGC0000354_c1 | | 12 |
| rh34 | NDR | NRPS | 9332 | NA | 202 | NA | | NA |
| rh34 | NDR | NRPS | 16090 | NA | 203 | NA | | NA |
| rh34 | NDR | NRPS | 38566 | Holomycin | 204 | BGC0000373_c1 | | 69 |
| rh34 | NDR | NRPS | 8072 | Pristinamycin | 205 | BGC0000952_c1 | | 7 |
| rh34 | NDR | NRPS | 11721 | Eponemycin | 206 | BGC0000345_c1 | | 14 |
| rh34 | NDR | Other | 22378 | SGR PTMs | 207 | BGC0001043_c1 | | 66 |
| rh34 | NDR | Other | 26167 | Glidobactin | 208 | BGC0000997_c1 | | 10 |
| rh34 | NDR | Other | 13722 | Echosides | 209 | BGC0000340_c1 | | 11 |
| rh34 | NDR | Other PKS | 8408 | Cosmomycin D | 210 | BGC0001074_c1 | | 5 |
| rh34 | NDR | Phenazine | 20275 | Endophenazines | 211 | BGC0000934_c1 | | 21 |
| rh34 | NDR | T2PKS | 4531 | Chartreusin | 212 | BGC0000206_c1 | | 9 |
| rh34 | NDR | T3PKS | 28233 | NA | 213 | NA | | NA |
| rh34 | NDR | T3PKS | 21317 | Alkylresorcinol | 214 | BGC0000282_c1 | | 100 |
| rh34 | NDR | Terpene | 12576 | NA | 215 | NA | | NA |
| rh34 | NDR | Terpene | 14380 | Isorenieratene | 216 | BGC0000664_c1 | | 28 |
| rh34 | NDR | Terpene | 8639 | Hopene | 217 | BGC0000663_c1 | | 38 |
| rh34 | NDR | Trans-AT PKS | 70084 | NA | 218 | NA | | NA |
| rh34 | NDR | NRPS | 7988 | NA | NA | NA | | NA |
| sk226 | INT | Butyrolactone-Ectoine | 15218 | Skyllamycin | 4 | BGC0000429_c1 | | 8 |
| sk226 | INT | Ectoine | 15822 | Ectoine | 6 | BGC0000853_c1 | | 100 |
| sk226 | INT | Melanin | 11771 | Melanin | 10 | BGC0000911_c1 | | 100 |
| sk226 | INT | T1PKS | 17563 | NA | 15 | NA | | NA |
| sk226 | INT | Siderophore | 11778 | Desferrioxamine B | 18 | BGC0000941_c1 | | 80 |
| sk226 | INT | Terpene | 16004 | Isorenieratene | 23 | BGC0000664_c1 | | 42 |
| sk226 | INT | Terpene | 9757 | NA | 25 | NA | | NA |
| sk226 | INT | Terpene | 24100 | NA | 28 | NA | | NA |
| sk226 | INT | Terpene | 18588 | Hopene | 30 | BGC0000663_c1 | | 69 |
| sk226 | INT | T3PKS | 16654 | Alkylresorcinol | 52 | BGC0000282_c1 | | 66 |
| sk226 | INT | Ladderane-Arylpolyene | 53778 | Skyllamycin | 59 | BGC0000429_c1 | | 24 |
| sk226 | INT | Lantipeptide | 11714 | AmfS | 61 | BGC0000496_c1 | | 40 |
| sk226 | INT | Melanin | 17306 | NA | 62 | NA | | NA |
| sk226 | INT | NRPS | 12950 | Viomycin | 64 | BGC0000458_c1 | | 42 |
| sk226 | INT | NRPS | 14836 | NA | 66 | NA | | NA |
| sk226 | INT | Other | 43146 | NA | 72 | NA | | NA |
| sk226 | INT | Siderophore | 8226 | NA | 76 | NA | | NA |
| sk226 | INT | Siderophore | 15283 | Kinamycin | 77 | BGC0000236_c1 | | 11 |
| sk226 | INT | T2PKS | 22415 | Griseorhodin | 78 | BGC0000230_c1 | | 60 |
| sk226 | INT | Thiopeptide-Lantipeptide | 26639 | NA | 82 | NA | | NA |
| sk226 | INT | NRPS | 11232 | Griseobactin | 95 | BGC0000368_c1 | | 29 |
| sk226 | INT | NRPS | 26192 | NA | 97 | NA | | NA |
| sk226 | INT | Bacteriocin | 2663 | NA | 219 | NA | | NA |
| sk226 | INT | Butyrolactone | 7624 | Coelimycin | 220 | BGC0000038_c1 | | 8 |
| sk226 | INT | Lantipeptide | 5718 | NA | 221 | NA | | NA |
| sk226 | INT | Lassopeptide | 7516 | SRO15-2005 | 222 | BGC0000578_c1 | | 60 |
| sk226 | INT | NRPS | 22079 | NA | 223 | NA | | NA |
| sk226 | INT | NRPS | 10834 | NA | 224 | NA | | NA |
| sk226 | INT | NRPS | 24517 | Viomycin | 225 | BGC0000458_c1 | | 57 |
| sk226 | INT | NRPS | 30107 | Streptolydigin | 226 | BGC0001046_c1 | | 5 |
| sk226 | INT | NRPS-T1PKS | 30149 | SGR PTMs | 227 | BGC0001043_c1 | | 100 |
| sk226 | INT | T1PKS | 9581 | Elaiophylin | 228 | BGC0000053_c1 | | 20 |
| sk226 | INT | T1PKS | 33545 | Himastatin | 229 | BGC0001117_c1 | | 12 |
| sk226 | INT | T3PKS | 23795 | NA | 230 | NA | | NA |
| sk226 | INT | T3PKS | 24492 | Herboxidiene | 231 | BGC0001065_c1 | | 3 |
| sk226 | INT | T1PKS | 2250 | NA | NA | NA | | NA |
| sk226 | INT | T1PKS | 3595 | NA | NA | NA | | NA |
| sk226 | INT | T1PKS | 1192 | NA | NA | NA | | NA |
| sk226 | INT | T1PKS | 1000 | NA | NA | NA | | NA |
| sk226 | INT | T1PKS | 9437 | Nigericin | NA | BGC0000114_c1 | | 50 |
| st115 | INT | Butyrolactone | 10960 | gamma-butyrolactone | 3 | BGC0000849_c1 | | 100 |
| st115 | INT | Ectoine | 8031 | Ectoine | 6 | BGC0000853_c1 | | 75 |
| st115 | INT | Lassopeptide | 22790 | SRO15-2005 | 9 | BGC0000578_c1 | | 60 |
| st115 | INT | Melanin | 11481 | Melanin | 10 | BGC0000911_c1 | | 100 |
| st115 | INT | NRPS | 25002 | Coelichelin | 12 | BGC0000325_c1 | | 72 |
| st115 | INT | NRPS | 35216 | SW-163 | 14 | BGC0000434_c1 | | 7 |
| st115 | INT | NRPS-T1PKS | 39131 | SGR PTMs | 16 | BGC0001043_c1 | | 100 |
| st115 | INT | Siderophore | 11778 | Desferrioxamine B | 18 | BGC0000941_c1 | | 80 |
| st115 | INT | Siderophore | 19661 | Kinamycin | 19 | BGC0000236_c1 | | 8 |
| st115 | INT | T3PKS | 31197 | Herboxidiene | 21 | BGC0001065_c1 | | 6 |
| st115 | INT | Terpene | 11383 | Steffimycin | 23 | BGC0000273_c1 | | 11 |
| st115 | INT | Terpene | 12384 | NA | 25 | NA | | NA |
| st115 | INT | Terpene | 22213 | NA | 28 | NA | | NA |
| st115 | INT | Terpene | 19466 | Hopene | 30 | BGC0000663_c1 | | 61 |
| st115 | INT | NRPS | 40829 | NA | 41 | NA | | NA |
| st115 | INT | NRPS | 4823 | NA | 63 | NA | | NA |
| st115 | INT | NRPS | 4663 | NA | 63 | NA | | NA |
| st115 | INT | NRPS | 9679 | NA | 69 | NA | | NA |
| st115 | INT | T3PKS | 2105 | NA | 79 | NA | | NA |
| st115 | INT | Thiopeptide-Lantipeptide | 10952 | NA | 82 | NA | | NA |
| st115 | INT | Bacteriocin | 9258 | Tetronasin | 83 | BGC0000163_c1 | | 3 |
| st115 | INT | NRPS | 18450 | NA | 97 | NA | | NA |
| st115 | INT | Other | 6390 | Pristinamycin | 100 | BGC0000952_c1 | | 5 |
| st115 | INT | Phenazine | 30146 | Phenazine | 102 | BGC0001080_c1 | | 38 |
| st115 | INT | NRPS-Other PKS | 61970 | Kirromycin | 172 | BGC0001070_c1 | | 8 |
| st115 | INT | T2PKS | 16113 | Fluostatin | 195 | BGC0000223_c1 | | 18 |
| st115 | INT | Bacteriocin | 5514 | NA | 232 | NA | | NA |
| st115 | INT | Butyrolactone-T2PKS | 12969 | Rabelomycin | 233 | BGC0000262_c1 | | 14 |
| st115 | INT | Ectoine | 10886 | Kosinostatin | 234 | BGC0001073_c1 | | 11 |
| st115 | INT | Ladderane-Arylpolyene | 6339 | Skyllamycin | 235 | BGC0000429_c1 | | 10 |
| st115 | INT | Lantipeptide | 15408 | NA | 236 | NA | | NA |
| st115 | INT | Lantipeptide | 8600 | NA | 237 | NA | | NA |
| st115 | INT | Lantipeptide | 21078 | Chalcomycin | 238 | BGC0000035_c1 | | 9 |
| st115 | INT | NRPS | 36893 | Daptomycin | 239 | BGC0000336_c1 | | 9 |
| st115 | INT | NRPS | 14133 | Balhimycin | 240 | BGC0000311_c1 | | 5 |
| st115 | INT | NRPS | 5364 | NA | 241 | NA | | NA |
| st115 | INT | NRPS | 21123 | Griseobactin | 242 | BGC0000368_c1 | | 64 |
| st115 | INT | Terpene | 25607 | Isorenieratene | 243 | BGC0000664_c1 | | 100 |
| st115 | INT | NRPS | 1647 | NA | NA | NA | | NA |
| st115 | INT | Other PKS | 20123 | SF2575 | NA | BGC0000269_c1 | | 4 |
| st140 | SDR | Bacteriocin | 9935 | NA | 1 | NA | | NA |
| st140 | SDR | Butyrolactone | 10944 | gamma-butyrolactone | 3 | BGC0000849_c1 | | 100 |
| st140 | SDR | Butyrolactone-Ectoine | 15163 | Skyllamycin | 4 | BGC0000429_c1 | | 8 |
| st140 | SDR | Other PKS-Butyrolactone | 47651 | SF2575 | 5 | BGC0000269_c1 | | 6 |
| st140 | SDR | Ectoine | 7423 | Ectoine | 6 | BGC0000853_c1 | | 100 |
| st140 | SDR | Lantipeptide | 23314 | SRO15-3108 | 7 | BGC0000554_c1 | | 100 |
| st140 | SDR | Lassopeptide | 22697 | SRO15-2005 | 9 | BGC0000578_c1 | | 80 |
| st140 | SDR | Melanin | 16058 | Melanin | 10 | BGC0000911_c1 | | 100 |
| st140 | SDR | NRPS | 28751 | Coelichelin | 12 | BGC0000325_c1 | | 81 |
| st140 | SDR | T1PKS | 13495 | NA | 15 | NA | | NA |
| st140 | SDR | Siderophore | 11778 | Desferrioxamine B | 18 | BGC0000941_c1 | | 80 |
| st140 | SDR | Siderophore | 29091 | Kinamycin | 19 | BGC0000236_c1 | | 20 |
| st140 | SDR | T2PKS | 25457 | Hedamycin | 20 | BGC0000233_c1 | | 59 |
| st140 | SDR | T3PKS | 41118 | Herboxidiene | 21 | BGC0001065_c1 | | 6 |
| st140 | SDR | Terpene | 11478 | Steffimycin | 23 | BGC0000273_c1 | | 11 |
| st140 | SDR | Terpene | 25278 | Isorenieratene | 24 | BGC0000664_c1 | | 71 |
| st140 | SDR | Terpene | 46314 | Stenothricin | 28 | BGC0000431_c1 | | 13 |
| st140 | SDR | Terpene | 20998 | Alnumycin | 29 | BGC0000195_c1 | | 9 |
| st140 | SDR | Terpene | 16764 | Hopene | 30 | BGC0000663_c1 | | 30 |
| st140 | SDR | Bacteriocin | 7114 | NA | 32 | NA | | NA |
| st140 | SDR | NRPS | 18059 | Enduracidin | 40 | BGC0000341_c1 | | 10 |
| st140 | SDR | T3PKS | 2903 | NA | 79 | NA | | NA |
| st140 | SDR | Terpene | 2979 | NA | 81 | NA | | NA |
| st140 | SDR | NRPS | 46293 | Kanamycin | 86 | BGC0000703_c1 | | 2 |
| st140 | SDR | NRPS-T1PKS | 33758 | SGR PTMs | 92 | BGC0001043_c1 | | 100 |
| st140 | SDR | Lantipeptide | 6698 | AmfS | 126 | BGC0000496_c1 | | 60 |
| st140 | SDR | NRPS | 18843 | Griseobactin | 244 | BGC0000368_c1 | | 41 |
| st140 | SDR | NRPS | 17985 | NA | 245 | NA | | NA |
| st140 | SDR | T1PKS | 37637 | NA | 246 | NA | | NA |
| st140 | SDR | T1PKS-Other PKS | 28699 | Hedamycin | 247 | BGC0000233_c1 | | 31 |
| st140 | SDR | Terpene | 9319 | Isorenieratene | 248 | BGC0000664_c1 | | 71 |
| st140 | SDR | Terpene | 20132 | NA | 249 | NA | | NA |
| st140 | SDR | Terpene-NRPS | 21865 | Griseoviridin | 250 | BGC0001097_c1 | | 10 |
| st170 | SDR | Butyrolactone | 10860 | gamma-butyrolactone | 3 | BGC0000849_c1 | | 100 |
| st170 | SDR | Ectoine | 8039 | Ectoine | 6 | BGC0000853_c1 | | 75 |
| st170 | SDR | Lassopeptide | 17442 | SRO15-2005 | 9 | BGC0000578_c1 | | 80 |
| st170 | SDR | Melanin | 8824 | Istamycin | 10 | BGC0000700_c1 | | 4 |
| st170 | SDR | NRPS-T1PKS | 32477 | Leinamycin | 15 | BGC0001101_c1 | | 4 |
| st170 | SDR | Siderophore | 11778 | Desferrioxamine B | 18 | BGC0000941_c1 | | 100 |
| st170 | SDR | Siderophore | 14717 | NA | 19 | NA | | NA |
| st170 | SDR | T3PKS | 34552 | Herboxidiene | 21 | BGC0001065_c1 | | 6 |
| st170 | SDR | Terpene | 11337 | Steffimycin | 23 | BGC0000273_c1 | | 11 |
| st170 | SDR | Terpene | 30412 | Isorenieratene | 24 | BGC0000664_c1 | | 85 |
| st170 | SDR | Terpene | 6319 | NA | 25 | NA | | NA |
| st170 | SDR | Terpene | 13905 | Hopene | 30 | BGC0000663_c1 | | 38 |
| st170 | SDR | Bacteriocin | 7826 | NA | 32 | NA | | NA |
| st170 | SDR | T3PKS | 1949 | NA | 79 | NA | | NA |
| st170 | SDR | Thiopeptide-Lantipeptide | 13882 | NA | 82 | NA | | NA |
| st170 | SDR | NRPS-T1PKS | 32890 | SGR PTMs | 92 | BGC0001043_c1 | | 100 |
| st170 | SDR | NRPS | 7377 | Griseobactin | 95 | BGC0000368_c1 | | 11 |
| st170 | SDR | Lantipeptide | 22903 | NA | 108 | NA | | NA |
| st170 | SDR | Butyrolactone | 10851 | Pristinamycin | 125 | BGC0000952 c3 | | 2 |
| st170 | SDR | Bacteriocin | 12964 | Enduracidin | 251 | BGC0000341_c1 | | 4 |
| st170 | SDR | Ectoine | 18244 | Pristinamycin | 252 | BGC0000952 c2 | | 23 |
| st170 | SDR | Lantipeptide | 22336 | AmfS | 253 | BGC0000496_c1 | | 100 |
| st170 | SDR | NRPS | 41642 | Zorbamycin | 254 | BGC0001058_c1 | | 10 |
| st170 | SDR | NRPS | 45160 | C-1027 | 255 | BGC0000965_c1 | | 53 |
| st170 | SDR | NRPS | 36809 | Bottromycin A2 | 256 | BGC0000469_c1 | | 9 |
| st170 | SDR | NRPS-T1PKS | 50303 | Cystothiazole A | 257 | BGC0000982_c1 | | 11 |
| st170 | SDR | NRPS | 10959 | C-1027 | 258 | BGC0000965_c1 | | 9 |
| st170 | SDR | NRPS | 23928 | A54145 | 259 | BGC0000291_c1 | | 3 |
| st170 | SDR | NRPS | 49055 | Griseobactin | 260 | BGC0000368_c1 | | 58 |
| st170 | SDR | NRPS-T1PKS | 15804 | A47934 | 261 | BGC0000290_c1 | | 5 |
| st170 | SDR | T1PKS | 33210 | C-1027 | 262 | BGC0000965_c1 | | 36 |
| st170 | SDR | Terpene-NRPS | 19035 | NA | 263 | NA | | NA |
| st170 | SDR | Terpene | 30784 | Isorenieratene | 264 | BGC0000664_c1 | | 85 |
| st77 | SDR | Bacteriocin | 13465 | NA | 1 | NA | | NA |
| st77 | SDR | Butyrolactone | 24343 | gamma-butyrolactone | 3 | BGC0000849_c1 | | 100 |
| st77 | SDR | Ectoine | 22384 | Ectoine | 6 | BGC0000853_c1 | | 100 |
| st77 | SDR | Lantipeptide | 17652 | AmfS | 8 | BGC0000496_c1 | | 80 |
| st77 | SDR | Lassopeptide | 20478 | SRO15-2005 | 9 | BGC0000578_c1 | | 80 |
| st77 | SDR | Melanin | 21916 | Istamycin | 10 | BGC0000700_c1 | | 4 |
| st77 | SDR | NRPS | 37565 | Coelichelin | 12 | BGC0000325_c1 | | 72 |
| st77 | SDR | NRPS | 29178 | Griseobactin | 13 | BGC0000368_c1 | | 47 |
| st77 | SDR | NRPS | 47342 | Phosphonoglycans | 14 | BGC0000806_c1 | | 3 |
| st77 | SDR | NRPS-T1PKS-Bacteriocin | 69342 | SGR PTMs | 16 | BGC0001043_c1 | | 100 |
| st77 | SDR | Siderophore | 11778 | Desferrioxamine B | 18 | BGC0000941_c1 | | 80 |
| st77 | SDR | Siderophore | 14764 | NA | 19 | NA | | NA |
| st77 | SDR | T3PKS | 41118 | Herboxidiene | 21 | BGC0001065_c1 | | 6 |
| st77 | SDR | Terpene | 21214 | Steffimycin | 23 | BGC0000273_c1 | | 19 |
| st77 | SDR | Terpene | 28707 | Isorenieratene | 24 | BGC0000664_c1 | | 85 |
| st77 | SDR | Terpene | 22108 | NA | 28 | NA | | NA |
| st77 | SDR | Terpene | 17099 | Alnumycin | 29 | BGC0000195_c1 | | 9 |
| st77 | SDR | Terpene | 17056 | Hopene | 30 | BGC0000663_c1 | | 61 |
| st77 | SDR | Terpene | 21232 | 2-methylisoborneol | 54 | BGC0000658_c1 | | 100 |
| st77 | SDR | Other | 6795 | NA | 100 | NA | | NA |
| st77 | SDR | NRPS | 10785 | Enduracidin | 176 | BGC0000341_c1 | | 4 |
| st77 | SDR | T3PKS | 37252 | Tetronasin | 214 | BGC0000163_c1 | | 11 |
| st77 | SDR | Terpene | 12449 | NA | 215 | NA | | NA |
| st77 | SDR | Arylpolyene | 41493 | Chloramphenicol | 265 | BGC0000893_c1 | | 11 |
| st77 | SDR | Lantipeptide | 12407 | NA | 266 | NA | | NA |
| st77 | SDR | Lantipeptide | 22858 | Labyrinthopeptin A1,A3/labyrinthopeptin A2 biosyntheticgene | 267 | BGC0000519_c1 | | 40 |
| st77 | SDR | NRPS | 57500 | Holomycin | 268 | BGC0000373_c1 | | 92 |
| st77 | SDR | NRPS | 56438 | Kedarcidin | 269 | BGC0000081_c1 | | 2 |
| st77 | SDR | NRPS-Ladderane | 55353 | Skyllamycin | 270 | BGC0000429_c1 | | 20 |
| st77 | SDR | NRPS-T1PKS | 34013 | Daptomycin | 271 | BGC0000336_c1 | | 4 |
| st77 | SDR | Other | 43614 | Neomycin | 272 | BGC0000710_c1 | | 8 |
| st77 | SDR | Phosphonate | 40836 | Dihydrochalcomycin | 273 | BGC0000047_c1 | | 6 |
| st77 | SDR | T1PKS-NRPS | 32974 | NA | 274 | NA | | NA |
| st77 | SDR | Terpene | 25573 | Isorenieratene | 275 | BGC0000664_c1 | | 100 |
| st77 | SDR | T1PKS | 1114 | NA | NA | NA | | NA |
| st77 | SDR | T1PKS | 1678 | NA | NA | NA | | NA |
| t99 | SDR | Bacteriocin | 11373 | NA | 1 | NA | | NA |
| t99 | SDR | Butyrolactone | 10369 | gamma-butyrolactone | 3 | BGC0000849_c1 | | 100 |
| t99 | SDR | Ectoine-Butyrolactone | 23805 | Pristinamycin | 4 | BGC0000952 c2 | | 23 |
| t99 | SDR | Butyrolactone-Other PKS | 32276 | SF2575 | 5 | BGC0000269_c1 | | 6 |
| t99 | SDR | Ectoine | 14731 | Ectoine | 6 | BGC0000853_c1 | | 100 |
| t99 | SDR | Lantipeptide | 14197 | SRO15-3108 | 7 | BGC0000554_c1 | | 75 |
| t99 | SDR | Lassopeptide | 22697 | SRO15-2005 | 9 | BGC0000578_c1 | | 80 |
| t99 | SDR | Melanin | 11918 | Istamycin | 10 | BGC0000700_c1 | | 4 |
| t99 | SDR | NRPS | 27448 | Coelichelin | 12 | BGC0000325_c1 | | 72 |
| t99 | SDR | NRPS | 29112 | Griseobactin | 13 | BGC0000368_c1 | | 41 |
| t99 | SDR | NRPS | 64506 | Phosphonoglycans | 14 | BGC0000806_c1 | | 3 |
| t99 | SDR | T1PKS | 13325 | NA | 15 | NA | | NA |
| t99 | SDR | Siderophore | 11778 | Desferrioxamine B | 18 | BGC0000941_c1 | | 100 |
| t99 | SDR | Siderophore | 24007 | Kinamycin | 19 | BGC0000236_c1 | | 11 |
| t99 | SDR | T1PKS-T2PKS | 34205 | Hedamycin | 20 | BGC0000233_c1 | | 59 |
| t99 | SDR | T3PKS | 34601 | Herboxidiene | 21 | BGC0001065_c1 | | 6 |
| t99 | SDR | Terpene | 13824 | Steffimycin | 23 | BGC0000273_c1 | | 11 |
| t99 | SDR | Terpene | 6258 | NA | 25 | NA | | NA |
| t99 | SDR | Terpene | 12720 | 2-methylisoborneol | 26 | BGC0000658_c1 | | 100 |
| t99 | SDR | Terpene | 25742 | Isorenieratene | 27 | BGC0000664_c1 | | 100 |
| t99 | SDR | Terpene | 22409 | NA | 28 | NA | | NA |
| t99 | SDR | Terpene | 14006 | Hopene | 30 | BGC0000663_c1 | | 30 |
| t99 | SDR | NRPS | 15695 | Enduracidin | 40 | BGC0000341_c1 | | 8 |
| t99 | SDR | T3PKS | 3295 | NA | 79 | NA | | NA |
| t99 | SDR | Bacteriocin | 10454 | NA | 83 | NA | | NA |
| t99 | SDR | NRPS-T1PKS | 32858 | SGR PTMs | 92 | BGC0001043_c1 | | 100 |
| t99 | SDR | NRPS-T1PKS | 32981 | Kanamycin | 96 | BGC0000703_c1 | | 2 |
| t99 | SDR | Other PKS | 8028 | Hedamycin | 101 | BGC0000233_c1 | | 18 |
| t99 | SDR | Lantipeptide | 18721 | NA | 199 | NA | | NA |
| t99 | SDR | NRPS | 15148 | NA | 245 | NA | | NA |
| t99 | SDR | Lantipeptide | 25452 | AmfS | 253 | BGC0000496_c1 | | 100 |
| t99 | SDR | T1PKS | 31865 | C-1027 | 276 | BGC0000965_c1 | | 3 |
| wa1002 | NDR | Butyrolactone | 12048 | Oxazolomycin | 3 | BGC0001106_c1 | | 6 |
| wa1002 | NDR | Ectoine | 5139 | Ectoine | 6 | BGC0000853_c1 | | 50 |
| wa1002 | NDR | Melanin | 5345 | Istamycin | 10 | BGC0000700_c1 | | 4 |
| wa1002 | NDR | NRPS | 25523 | Coelichelin | 12 | BGC0000325_c1 | | 81 |
| wa1002 | NDR | T1PKS | 16852 | NA | 15 | NA | | NA |
| wa1002 | NDR | Siderophore | 20305 | Kinamycin | 19 | BGC0000236_c1 | | 8 |
| wa1002 | NDR | T3PKS | 25271 | Herboxidiene | 21 | BGC0001065_c1 | | 6 |
| wa1002 | NDR | Terpene | 12843 | Steffimycin | 23 | BGC0000273_c1 | | 11 |
| wa1002 | NDR | Terpene | 18747 | NA | 28 | NA | | NA |
| wa1002 | NDR | Terpene | 13797 | Hopene | 30 | BGC0000663_c1 | | 30 |
| wa1002 | NDR | NRPS-Ladderane-Arylpolyene | 46478 | Skyllamycin | 31 | BGC0000429_c1 | | 28 |
| wa1002 | NDR | Bacteriocin | 7574 | NA | 32 | NA | | NA |
| wa1002 | NDR | Butyrolactone | 11833 | Lactonamycin | 33 | BGC0000238_c1 | | 3 |
| wa1002 | NDR | Lassopeptide | 24063 | NA | 36 | NA | | NA |
| wa1002 | NDR | NRPS | 12502 | Enduracidin | 40 | BGC0000341_c1 | | 10 |
| wa1002 | NDR | NRPS | 30212 | Arylomycin | 43 | BGC0000306_c1 | | 22 |
| wa1002 | NDR | NRPS-T3PKS-Ectoine | 58208 | Lobophorin | 45 | BGC0001183_c1 | | 10 |
| wa1002 | NDR | T1PKS | 35518 | Calicheamicin | 46 | BGC0000033_c1 | | 10 |
| wa1002 | NDR | Other PKS | 20840 | Lactonamycin | 47 | BGC0000238_c1 | | 14 |
| wa1002 | NDR | Siderophore | 7628 | Desferrioxamine B | 48 | BGC0000941_c1 | | 100 |
| wa1002 | NDR | T2PKS | 31886 | Kiamycin | 50 | BGC0000235_c1 | | 80 |
| wa1002 | NDR | T2PKS | 22069 | Macrotetrolide | 51 | BGC0000243_c1 | | 100 |
| wa1002 | NDR | T3PKS | 20965 | Galbonolides | 53 | BGC0000065_c1 | | 6 |
| wa1002 | NDR | Terpene | 15261 | 2-methylisoborneol | 54 | BGC0000658_c1 | | 100 |
| wa1002 | NDR | Thiopeptide | 26233 | Rabelomycin | 56 | BGC0000262_c1 | | 10 |
| wa1002 | NDR | NRPS | 4281 | NA | 63 | NA | | NA |
| wa1002 | NDR | T3PKS | 2081 | NA | 79 | NA | | NA |
| wa1002 | NDR | Lantipeptide | 4104 | AmfS | 126 | BGC0000496_c1 | | 40 |
| wa1002 | NDR | Melanin | 11815 | Melanin | 150 | BGC0000912_c1 | | 100 |
| wa1002 | NDR | Other | 14365 | Tomaymycin | 170 | BGC0000448_c1 | | 23 |
| wa1002 | NDR | Terpene | 5422 | NA | 187 | NA | | NA |
| wa1002 | NDR | NRPS | 5863 | NA | 241 | NA | | NA |
| wa1002 | NDR | NRPS | 3326 | NA | 241 | NA | | NA |
| wa1002 | NDR | Ectoine | 14884 | Ectoine | 277 | BGC0000853_c1 | | 75 |
| wa1002 | NDR | Lassopeptide | 20997 | NA | 278 | NA | | NA |
| wa1002 | NDR | NRPS | 2494 | NA | 279 | NA | | NA |
| wa1002 | NDR | NRPS | 7997 | NA | 280 | NA | | NA |
| wa1002 | NDR | NRPS | 28476 | NA | 281 | NA | | NA |
| wa1002 | NDR | NRPS | 3332 | NA | 282 | NA | | NA |
| wa1002 | NDR | NRPS-T1PKS | 21048 | SGR PTMs | 283 | BGC0001043_c1 | | 100 |
| wa1002 | NDR | Other | 17626 | Collismycin A | 284 | BGC0000973_c1 | | 29 |
| wa1002 | NDR | T1PKS-NRPS | 11808 | NA | 285 | NA | | NA |
| wa1002 | NDR | NRPS | 2666 | NA | NA | NA | | NA |
| wa1002 | NDR | NRPS | 3193 | NA | NA | NA | | NA |
| wa1063 | NDR | Butyrolactone | 10641 | Oxazolomycin | 3 | BGC0001106_c1 | | 6 |
| wa1063 | NDR | Ectoine | 5811 | Ectoine | 6 | BGC0000853_c1 | | 50 |
| wa1063 | NDR | Terpene | 13506 | NA | 28 | NA | | NA |
| wa1063 | NDR | Terpene | 10714 | Phosphonoglycans | 30 | BGC0000807_c1 | | 6 |
| wa1063 | NDR | NRPS-Ladderane-Arylpolyene | 76981 | Skyllamycin | 31 | BGC0000429_c1 | | 36 |
| wa1063 | NDR | Bacteriocin | 7382 | NA | 32 | NA | | NA |
| wa1063 | NDR | Butyrolactone | 12946 | Coelimycin | 33 | BGC0000038_c1 | | 16 |
| wa1063 | NDR | Lassopeptide | 14995 | NA | 37 | NA | | NA |
| wa1063 | NDR | Other PKS | 20924 | Lactonamycin | 47 | BGC0000238_c1 | | 14 |
| wa1063 | NDR | Siderophore | 10232 | Desferrioxamine B | 48 | BGC0000941_c1 | | 100 |
| wa1063 | NDR | T2PKS | 37028 | Kiamycin | 50 | BGC0000235_c1 | | 95 |
| wa1063 | NDR | T3PKS | 19443 | NA | 53 | NA | | NA |
| wa1063 | NDR | Terpene | 16562 | 2-methylisoborneol | 54 | BGC0000658_c1 | | 100 |
| wa1063 | NDR | Lantipeptide | 7635 | NA | 61 | NA | | NA |
| wa1063 | NDR | Other | 6995 | NA | 100 | NA | | NA |
| wa1063 | NDR | Siderophore | 9276 | NA | 103 | NA | | NA |
| wa1063 | NDR | NRPS | 14443 | Coelichelin | 128 | BGC0000325_c1 | | 18 |
| wa1063 | NDR | Melanin | 6256 | Melanin | 150 | BGC0000912_c1 | | 100 |
| wa1063 | NDR | Other | 8770 | Tomaymycin | 170 | BGC0000448_c1 | | 17 |
| wa1063 | NDR | NRPS | 7894 | NA | 176 | NA | | NA |
| wa1063 | NDR | Terpene | 5255 | NA | 187 | NA | | NA |
| wa1063 | NDR | Ectoine | 17727 | Ectoine | 277 | BGC0000853_c1 | | 50 |
| wa1063 | NDR | NRPS | 3404 | NA | 279 | NA | | NA |
| wa1063 | NDR | NRPS | 11795 | NA | 285 | NA | | NA |
| wa1063 | NDR | Lantipeptide | 1879 | NA | 286 | NA | | NA |
| wa1063 | NDR | Lantipeptide | 23376 | NA | 287 | NA | | NA |
| wa1063 | NDR | Lassopeptide | 2162 | NA | 288 | NA | | NA |
| wa1063 | NDR | Melanin | 2894 | Istamycin | 289 | BGC0000700_c1 | | 4 |
| wa1063 | NDR | NRPS | 5482 | NA | 290 | NA | | NA |
| wa1063 | NDR | NRPS | 4425 | NA | 291 | NA | | NA |
| wa1063 | NDR | NRPS | 7989 | NA | 292 | NA | | NA |
| wa1063 | NDR | NRPS | 14393 | SGR PTMs | 293 | BGC0001043_c1 | | 83 |
| wa1063 | NDR | NRPS | 21405 | Lobophorin | 294 | BGC0001183_c1 | | 3 |
| wa1063 | NDR | NRPS-Ectoine-T3PKS | 26725 | Lobophorin | 295 | BGC0001183_c1 | | 8 |
| wa1063 | NDR | Other | 1842 | NA | 296 | NA | | NA |
| wa1063 | NDR | T2PKS | 9643 | Macrotetrolide | 297 | BGC0000243_c1 | | 50 |
| wa1063 | NDR | T3PKS | 2027 | NA | 298 | NA | | NA |
| wa1063 | NDR | T3PKS | 13547 | Herboxidiene | 299 | BGC0001065_c1 | | 5 |
| wa1063 | NDR | NRPS | 2116 | NA | NA | NA | | NA |
| wa1063 | NDR | Other | 1485 | NA | NA | NA | | NA |
| wa1063 | NDR | Other | 1039 | NA | NA | NA | | NA |
| wa1063 | NDR | T1PKS | 6000 | Stambomycin | NA | BGC0000151_c1 | | 36 |
| wa1063 | NDR | T1PKS | 5871 | ECO-02301 | NA | BGC0000052_c1 | | 32 |
| wa1071 | NDR | Ectoine | 5811 | Ectoine | 6 | BGC0000853_c1 | | 50 |
| wa1071 | NDR | Siderophore | 19541 | Kinamycin | 19 | BGC0000236_c1 | | 5 |
| wa1071 | NDR | T3PKS | 20846 | Herboxidiene | 21 | BGC0001065_c1 | | 6 |
| wa1071 | NDR | Terpene | 13945 | Steffimycin | 23 | BGC0000273_c1 | | 11 |
| wa1071 | NDR | Terpene | 9930 | NA | 28 | NA | | NA |
| wa1071 | NDR | Terpene | 9924 | Hopene | 30 | BGC0000663_c1 | | 23 |
| wa1071 | NDR | NRPS-Ladderane-Arylpolyene | 63073 | Skyllamycin | 31 | BGC0000429_c1 | | 32 |
| wa1071 | NDR | Butyrolactone | 11833 | Lactonamycin | 33 | BGC0000238_c1 | | 3 |
| wa1071 | NDR | Siderophore | 7624 | Desferrioxamine B | 48 | BGC0000941_c1 | | 100 |
| wa1071 | NDR | T2PKS | 20288 | Rabelomycin | 50 | BGC0000262_c1 | | 22 |
| wa1071 | NDR | Terpene | 16413 | 2-methylisoborneol | 54 | BGC0000658_c1 | | 100 |
| wa1071 | NDR | Lantipeptide | 8522 | NA | 61 | NA | | NA |
| wa1071 | NDR | NRPS | 2899 | NA | 65 | NA | | NA |
| wa1071 | NDR | T3PKS | 2468 | NA | 79 | NA | | NA |
| wa1071 | NDR | Other | 6888 | NA | 100 | NA | | NA |
| wa1071 | NDR | Terpene | 3066 | NA | 107 | NA | | NA |
| wa1071 | NDR | NRPS | 4468 | NA | 132 | NA | | NA |
| wa1071 | NDR | Melanin | 9395 | Melanin | 150 | BGC0000912_c1 | | 100 |
| wa1071 | NDR | Other | 8027 | Tomaymycin | 170 | BGC0000448_c1 | | 11 |
| wa1071 | NDR | Terpene | 5388 | NA | 187 | NA | | NA |
| wa1071 | NDR | Bacteriocin | 3365 | NA | 196 | NA | | NA |
| wa1071 | NDR | NRPS | 2031 | NA | 241 | NA | | NA |
| wa1071 | NDR | Ectoine | 15534 | Ectoine | 277 | BGC0000853_c1 | | 75 |
| wa1071 | NDR | Lassopeptide | 18071 | NA | 278 | NA | | NA |
| wa1071 | NDR | Other | 18937 | Collismycin A | 284 | BGC0000973_c1 | | 29 |
| wa1071 | NDR | NRPS | 11148 | NA | 285 | NA | | NA |
| wa1071 | NDR | Lantipeptide | 2040 | NA | 286 | NA | | NA |
| wa1071 | NDR | NRPS | 2881 | NA | 291 | NA | | NA |
| wa1071 | NDR | NRPS-T1PKS | 11367 | NA | 292 | NA | | NA |
| wa1071 | NDR | NRPS | 13699 | SGR PTMs | 293 | BGC0001043_c1 | | 83 |
| wa1071 | NDR | NRPS-Ectoine-T3PKS | 28653 | Lobophorin | 295 | BGC0001183_c1 | | 8 |
| wa1071 | NDR | Butyrolactone | 5748 | NA | 300 | NA | | NA |
| wa1071 | NDR | Lassopeptide | 5117 | NA | 301 | NA | | NA |
| wa1071 | NDR | Melanin | 1571 | Istamycin | 302 | BGC0000700_c1 | | 4 |
| wa1071 | NDR | NRPS | 13951 | NA | 303 | NA | | NA |
| wa1071 | NDR | NRPS | 8736 | Coelichelin | 304 | BGC0000325_c1 | | 27 |
| wa1071 | NDR | NRPS | 13371 | Coelichelin | 305 | BGC0000325_c1 | | 63 |
| wa1071 | NDR | Other | 11852 | Cycloheximide/actiphenol | 306 | BGC0000175_c1 | | 33 |
| wa1071 | NDR | Other PKS | 8817 | Coelimycin | 307 | BGC0000038_c1 | | 8 |
| wa1071 | NDR | T1PKS | 19754 | Streptolydigin | 308 | BGC0001046_c1 | | 15 |
| wa1071 | NDR | T1PKS | 3325 | NA | 309 | NA | | NA |
| wa1071 | NDR | T2PKS | 2141 | NA | 310 | NA | | NA |
| wa1071 | NDR | NRPS | 2841 | NA | NA | NA | | NA |
| wa1071 | NDR | Other | 1086 | NA | NA | NA | | NA |
| wa1071 | NDR | Other | 1730 | NA | NA | NA | | NA |
| wa1071 | NDR | Other | 1734 | NA | NA | NA | | NA |
| wa1071 | NDR | Other | 4164 | NA | NA | NA | | NA |
